# Supplementary material for: Reconfiguring nucleation for CVD growth of twisted bilayer MoS2 with a wide range of twist angles
Source: Nat Commun. 2024 Jan 17;15:562. doi: 10.1038/s41467-023-44598-w (PMC10794196; doi:10.1038/s41467-023-44598-w)
Supplement: Supplementary file 1 — Supplementary Information [file 41467_2023_44598_MOESM1_ESM.pdf]

Supplementary Information

Reconfiguring nucleation for CVD growth of twisted bilayer MoS_2 with a wide range of twist angles

Manzhang Xu[1,2,3,‡], Hongjia Ji[1,‡], Lu Zheng[1,2,3], Weiwei Li[1,2,3], Jing Wang[4], Hanxin Wang[1], Lei Luo[1], Qianbo Lu[1,2,3], Xuetao Gan[4], Zheng Liu[5,6,7], Xuewen Wang[1,2,3,*], Wei Huang[1,2,3,8,9,*]

[1] Frontiers Science Center for Flexible Electronics (FSCFE) & Institute of Flexible Electronics (IFE), Northwestern Polytechnical University, Xi'an 710072, P. R. China.

[2] MIIT Key Laboratory of Flexible Electronics (KLoFE), Northwestern Polytechnical University, Xi'an 710072, P. R. China.

[3] Shaanxi Key Laboratory of Flexible Electronics (KLoFE), Northwestern Polytechnical University, Xi'an, 710072, P. R. China.

[4] Key Laboratory of Light Field Manipulation and Information Acquisition, Ministry of Industry and Information Technology, and Shaanxi Key Laboratory of Optical Information Technology, School of Physical Science and Technology, Northwestern Polytechnical University, Xi'an 710129, P. R. China.

[5] School of Materials Science and Engineering, Nanyang Technological University, 50 Nanyang Avenue, Singapore 639798, Singapore.

[6] CINTRA CNRS/NTU/THALES, UMI 3288, Research Techno Plaza, 50 Nanyang Drive, Border X Block, Level 6, Singapore 637553, Singapore.

[7] School of Electrical and Electronic Engineering, Nanyang Technological University, Singapore 639798, Singapore.

[8] State Key Laboratory of Organic Electronics and Information Displays, Institute of Advanced Materials (IAM), Nanjing University of Posts & Telecommunications, Nanjing, 210023, P. R. China.

[9] Key Laboratory of Flexible Electronics (KloFE) and Institute of Advanced Materials (IAM), Nanjing Tech University (NanjingTech), Nanjing, 211800, P. R. China.

‡ These authors contributed equally to this work.

* Corresponding author: Xuewen Wang: iamxwwang@nwpu.edu.cn, Wei Huang: vc@nwpu.edu.cn.

List of Notes

Supplementary Note 1. Characterization of the twist angle based on OM.

Supplementary Note 2. Characterizing the twist angle based on SAED.

Supplementary Note 3. Numerical simulations of the field velocity distribution, temperature distribution, and gas flow patterns.

Supplementary Note 4. The density and yield calculation of TB-MoS_2.

Supplementary Note 5. Thermodynamically and kinetics analysis of NaCl.

Supplementary Note 6. Kinetics analysis of confined space.

List of Tables

Supplementary Table 1. Summary of the CVD preparation of the TB-TMDCs.

List of Figures

Supplementary Fig. 1 Rules for measuring the twist angles and corresponding error analysis of TB-MoS_2 based on OM method.

Supplementary Fig. 2 The BF-OM, DF-OM and the corresponding polar plots of parallel components of SHG intensity of TB-MoS_2.

Supplementary Fig. 3 The relationship between the SHG azimuthal angle and twist angle measured from OM in TB-MoS_2.

Supplementary Fig. 4 BF-OM and DF-OM images of as-grown TB-MoS_2.

Supplementary Fig. 5 Characterization of 21.9°-TB-MoS_2.

Supplementary Fig. 6 BF-OM image of 101.3°-TB-MoS_2 for Raman and PL mapping.

Supplementary Fig. 7 BF-OM image of 90°-TB-MoS_2 for AFM and high-resolution AFM measurement.

Supplementary Fig. 8 The twist angle measurement of 90°-TB-MoS_2 based on the FFT from high-resolution LFM images.

Supplementary Fig. 9 SAED patterns with diffracted intensity of TB-MoS_2.

Supplementary Fig. 10 Error analysis of twist angle measurement based on SAED method.

Supplementary Fig. 11 The twist angle measurement of 21.9°-TB-MoS_2.

Supplementary Fig. 12 HAADF-STEM results of TB-MoS_2.

Supplementary Fig. 13 The atom structure of MoS_2.

Supplementary Fig. 14 Velocity distribution of the space-confined CVD setup under different gas flow rates.

Supplementary Fig. 15 OM of TB-MoS_2 for repeated synthesis in three times.

Supplementary Fig. 16 OM of CVD synthesized large-area TB-MoS_2 on SiO_2/Si under the 5X objective.

Supplementary Fig. 17 Typical BF-OM of the synthesized products under different reaction temperatures.

Supplementary Fig. 18 Statistical distribution of twist angles under different growth conditions.

Supplementary Fig. 19 OM of synthesized 1.2° and 58.2°-TB-MoS_2.

Supplementary Fig. 20 Frequency distribution histograms of flake size of TB-MoS_2 and corresponding gamma distribution fitting curve (with the mean value (E) and standard deviation (σ)).

Supplementary Fig. 21 Typical BF-OM of synthesized products under different gas flow rates.

Supplementary Fig. 22 Typical BF-OM of synthesized products under different molar ratios of NaCl to MoO_3.

Supplementary Fig. 23 Typical BF-OM of synthesized products with/without NaCl and confined space.

Supplementary Fig. 24 The typical OM and Raman spectrum of the products without adding of NaCl.

Supplementary Fig. 25 The typical BF-OM of products synthesized under different salt ratio.

Supplementary Fig. 26 Proportion of monolayer MoS_2, bilayer MoS_2, multi-layer MoS_2, and non-MoS_2 in the as-grown samples.

Supplementary Fig. 27 Velocity distribution, temperature distribution, and velocity vector maps of the CVD setup.

Supplementary Fig. 28 Characterization of TB-WS_2.

Supplementary Notes

Supplementary Note 1. Characterization of the twist angle based on OM.

The TB-MoS_2 shows the triangle morphology in this work, and the monolayer and bilayer share the same nucleation site. Therefore, an edge of the monolayer (dashed green line) rotates counterclockwise to meet one side of the bilayer (dashed red line). The rotated angle is defined as the twist angle θ (0°<θ<120°) (Supplementary Fig. 1a). Measuring the edges of MoS_2 to estimate the twist angle is an efficient way to calculate the twist angles of all bilayer-MoS_2. Here, we summarized three types of TB-MoS_2 morphologies and the corresponding rules to measure the twist angles, as shown in Supplementary Fig. 1a-c.

The twist angles of the TB-MoS_2 can be directly measured based on the regular triangles from the OM when the triangle with regular edge (Type I). Three twist angles can be measured and the average values are calculated (Supplementary Fig. 1a). The triangle surrounded by satellite-triangle (Type II) samples should be measured by the largest triangle (Supplementary Fig. 1b). For the triangle with irregular edges, the triangle can be replaced by the external inscribed regular triangle for the twist angle measurement of TB-MoS_2 (Supplementary Fig. 1c). The average of six twist angles (with three θ_{out} and three θ_{in}) is considered to be the twist angle of TB-MoS_2.

Here, a TB-MoS_2 sample was used to calculate the twisted angle by OM. As shown in Supplementary Fig. 1b, the twist angle can be measured to be 22.0°, 21.5°, and 22.4°. The average value of 22.0° with a standard deviation of about 0.35° was used as the twist angle (Supplementary Fig. 1d). Actually, the error is inevitable when measuring the orientations of each edge (Supplementary Fig. 1f) due to the limited resolution (the minimum resolution σ for an optical microscope is around 200 nm, which responds to the size of fuzzy region) of OM images. The errors (φ_{OM}) of each edge can be determined as:

$$\varphi_{\mathrm{OM}} = \arctan\frac{\sigma}{L} \tag{1}$$

where L is the length of the triangle, σ is the minimum resolution of OM. Here, we can notice that the φ_{OM} is dependent on the length of the triangle, thus we use the average length of TB-MoS_2 to calculate the error. The average L is calculated to be 28.56 μm (Supplementary Fig. 20) and the error (φ_{OM}) is 0.4°. Due to the twist angle (θ) being determined by measuring two edges of the monolayer and bilayer, the OM based error of the twist angle should be twice the value of the error (φ_{OM}):

$$\mathrm{Error}_{\theta-\mathrm{OM}}=2\times\varphi_{\mathrm{OM}}\approx 0.8^{\circ} \quad (2)$$

As shown in Supplementary Fig. 1, the standard deviation is about 0.35°, smaller than the $\mathrm{Error}_{\theta-\mathrm{OM}}$ of 0.8°.

Supplementary Note 2. Characterizing the twist angle based on SAED.

The SAED is an accurate method for measuring the twist angle of TB-MoS_2, which is widely used to determine the twist angle of 2D materials, such as TB-graphene and TB-TMDCs. Firstly, based on the HAADF-STEM image of TB-MoS_2, we can preliminarily identify the twist angle θ_{STEM} range belonging to 0°~30°, 30°~60°, 60°~90°, or 90°~120°. Then, we measure the direction of two opposite diffraction points of each layer MoS_2, and the twist angle θ_{SAED} can be measured in the range of 0° to 30°. Based on the following equations, the twist angle θ_{STEM} can be finally determined.

$$\theta_{\mathrm{SAED}}=\theta_{\mathrm{STEM}}\left(0<\theta_{\mathrm{STEM}}\leq 30°\right) \tag{3}$$

$$\theta_{\mathrm{SAED}}=60°-\theta_{\mathrm{STEM}}\ \left(30°<\theta_{\mathrm{STEM}}\leq 60°\right) \tag{4}$$

$$\theta_{\mathrm{SAED}}=\theta_{\mathrm{STEM}}-60°\ \left(60°<\theta_{\mathrm{STEM}}\leq 90°\right) \tag{5}$$

$$\theta_{\mathrm{SAED}}=120°-\theta_{\mathrm{STEM}}\left(90°<\theta_{\mathrm{STEM}}\leq 120°\right) \tag{6}$$

Based on the measurement, the direction of two opposite diffraction points of each MoS_2 layer is much higher than the OM method as discussed in Supplementary Note 1. Taking a SAED pattern of 21.9°-TB- MoS_2 as an example (as shown Supplementary Fig. 10a), the weight lines connecting the opposite diffraction points intersect with each other at a specific twist angle.

The error (φ_{SAED}) based on the SAED pattern can be determined by the full width at half maximum (FWHM) of the blurred diffraction points and the distance between each opposite bright point in a single SAED pattern (Supplementary Fig. 10b).

$$\varphi_{\mathrm{SAED}i}=\arctan\frac{FWHM_i}{D_i} \tag{7}$$

where i is the diffraction order ($i=1,2,3,4...$), D_i is the distance from the two opposite bright points under the diffraction order of i. In order to calculate the error based on the SAED method, we measure FWHMs of four groups of the position dependent diffraction intensity

plot (1st, 2nd, 3rd, and 5th, Supplementary Fig. 10c-f) with the average FWHMs of 0.0049 nm^{-1}. While the distance from the two opposite bright point with the diffraction order i can be measured to be (D_1 = 7.356 nm^{-1}, D_2 =12.542 nm^{-1}, D_3 =14.666 nm^{-1}, and D_5 =21.986 nm^{-1}), indicating that the error will be smaller if higher order diffraction points are selected. Thus, the error of twist angle ($\mathrm{Error}_{\theta\text{-SAED}}$) should be the twice of the value of $\varphi_{\mathrm{SAED}i}$ due to the measurement for two times. In our work, the 2nd diffraction point is selected, and the $\mathrm{Error}_{\theta\text{-SAED}}$ is calculated to be 0.44°, which is more accurate than the $\mathrm{Error}_{\theta\text{-OM}}$ of 0.8°.

$$\mathrm{Error}_{\theta\text{-SAED}} = 2 \times \varphi_{\mathrm{SAED2}} \approx 0.44° \quad (8)$$

Supplementary Note 3. Numerical simulations of the field velocity distribution, temperature distribution, and gas flow patterns.

Numerical simulations of the field velocity distribution, temperature distribution, and gas flow patterns were conducted with the finite volume method (FVM) using commercial software ANSYS Fluent. A 2D model was exploited with a mesh size of 1 mm × 1 mm, which is one-tenth of the diameter of the inner tube. In our simulation, the gases were treated as a mixture and the flow was assumed to be steady. The boundary condition was set as: the size of the quartz tube (1200 mm × 25.4 mm), the size of the inner tube (100 mm × 12 mm), the length of constant temperature region (200 mm), temperature of constant temperature zone (high temperature range, 780 ˚C), the temperature of nonconstant temperature zone (Room temperature, 25 ˚C), the gas pressure in the tube (determined by the gas flow rate), and the gas pressure out of the tube (atmospheric pressure).

Supplementary Note 4. The density and yield calculation of TB-MoS_2.

The OM under the 20X objective was selected due to the TB-MoS_2 can be easily identified under the 20X objective. The number of TB-MoS_2 and bilayer MoS_2 based on the OM under the 20X objective was calculated. The density (D_{TB}) and yield (Y_{TB}) can be calculated by the total number of TB-MoS_2 samples (N_{TB}), the total number of bilayer MoS_2 samples (N_{BL}), and the total area (S).

$$D_{TB} = \frac{N_{TB}}{S} \qquad (9)$$

$$Y_{TB} = \frac{N_{TB}}{N_{BL}} \times 100\% \qquad (10)$$

To make the results more accurate, five samples were adopted under the same growth conditions. The average density ($\overline{D}$), standard deviation (δ_D, density error), yield ($\overline{Y}$), and corresponding standard deviation (δ_Y, yield error)were canulated by the following equations.

$$\overline{D} = \frac{\sum_{i=1}^{5} D_{TBi}}{5}, \delta_D = \sqrt{\frac{\sum_{i=1}^{5}\left(D_i - \overline{D}\right)}{5}} \qquad (11)$$

$$\overline{Y} = \frac{\sum_{i=1}^{5} Y_{TBi}}{5}, \delta_Y = \sqrt{\frac{\sum_{i=1}^{5}\left(Y_i - \overline{Y}\right)}{5}} \qquad (12)$$

Supplementary Note 5. Thermodynamically and kinetics analysis of NaCl.

In our previous works, we have discussed the effects of salt on the CVD growth of TMDCs via thermodynamics and kinetics [1, 2]. In terms of affecting thermodynamics, NaCl can be reacted with MoO_3 for the synthesis of MoO_xCl_y in first step. Notably, the degrees of freedom changed by reacting with MoO_xCl_y results in the change of entropy. Compared with the CVD system without NaCl, the participation of MoO_xCl_y may affect the Gibbs free energy ($\Delta G = \Delta H - T\Delta S$) by neglecting the change of pressure and volume. For the kinetics process, NaCl will affect the nucleation process and dominate the geometries of MoS_2 layers. The nucleation rate with and without NaCl can be roughly written as:

$$\frac{\dot{N}\left(MoO_3 + NaCl\right)}{\dot{N}\left(MoO_3\right)} \sim \frac{P_i\left(NaCl\right)}{P_i\left(without\ \ NaCl\right)} \qquad (13)$$

From this equation, we can find that the partial pressure P_i is the dominate factor for the nucleation rate. As we mentioned before [1], the vapor pressure of NaCl is much higher than that of MoO_3 precursors. Besides, the formation of MoO_xCl_y induces a high P_i due to the high volatility nature of MoO_xCl_y. Therefore, the NaCl will result in a higher nucleation rate. The vapor pressure of NaCl and MoO_3 can be found to be around ~10 Pa and ~10^{-9} Pa under 780 ° C (1053 K), respectively. Considering that the solubility of metal oxide is at the order of ppm, the metal oxide and salt will dramatically increase the vapor pressure of metal precursors of a few orders.

Supplementary Note 6. Kinetics analysis of confined space.

To further clarify the effect of the confined space, we have carried out the computational fluid dynamics simulations of the confined space in CVD. The velocity distribution, temperature distribution, and components of the velocity vector for this CVD setup are shown in Supplementary Fig. 27. Here, we believe that the confined space will change the gas flow rate, the temperature, and the gas flow direction. The corresponding detail discussion are shown below.

(1) Gas flow rate.

It is observed from the velocity distribution that the gas flow condition affects the sample state in the open tube system (Supplementary Fig. 14 and Supplementary Fig. 27). It can be identified that the gas velocity in the inner tube (without sealed-end) is one order of magnitude less than that of the outer tube, indicating that there is a change in the gas fluid velocity on the SiO_2/Si substrate. With the decrease in gas flow rate, the fluid velocity of carrier gas decreases, making the nuclei surplus. As a result, the nuclei will grow larger, which is consistent with the experimental results (Supplementary Fig. 21). As we know, small change in the flow regime in CVD significantly influences the reaction products. Although the gas velocity in the inner tube has been greatly reduced, there is a nonnegligible increase. Therefore, there is a rapid change in the yield and density of TB-MoS_2, indicating that the gas flow rate is important in the synthesis process, which mainly affects the nucleation and growth of TB-MoS_2.

(2) Temperature.

Temperature is another key parameter for CVD process, wherein the different reaction temperatures may result in different morphology [3-6]. Based on previous reports, the preparation of MoS_2 usually requires a relatively high reaction temperature from 600 °C to 1000 °C [1, 4, 7]. However, based on our experimental results, we find that the TB-MoS_2 can only be well synthesized under a narrow reaction temperature range from 770 °C to 790 °C. As shown in Supplementary Fig. 17, TB-MoS_2 can be obtained under a reaction temperature of 780 °C. Higher or lower temperatures result in the dramatic decrease in the yield of TB-MoS_2 For

example, large-area monolayer MoS_2 is synthesized under a reaction temperature of 760 °C.

Temperature is the key to affect the thermodynamics and dynamics of the reaction system. Here, we have carried out the simulation on the temperature in the tube, as shown in Supplementary Fig. 27c-d. As noticed, due to the effect of the gas flow rate, the temperature shows an uneven distribution. However, with the introduction of the confined space, there is a uniform temperature distribution near the SiO_2/Si substrate. The inner tube serves as a "heat insulation layer" to prevent the heat diffusion. Thus, we believe that the confined space plays the key role to stabilize the substrate temperature.

(3) Turbulent flow.

In most cases, laminar gas flow is desirable, but some local areas of turbulent flow may exist. Generally, conventional CVD that uses tubular horizontal reactors possesses laminar flow under any conditions. A high pressure or flow rate is required to produce turbulent flow in the reactor, which exceeds the flow rate used in CVD. To induce turbulent flow, some form of physical disturbance of the gas flow is needed in the CVD setup. In this work, we introduce the confined space to CVD to induce the turbulent flow in the inner tube. This design creates a circumfluent flow, wherein the gas flows around the chamber and backflows toward the substrate. This significantly reduces the gas flow velocity on the substrate to create an unsteady gas flow.

It is reported that the turbulent flow indeed causes the different growth conditions in CVD process. The turbulent flow has been adopted for the synthesis of 2D materials, such as graphene and TMDCs, which effectively changes the morphology of the products [8-10]. The components of the velocity vector V_x, V_y, and V_z in this confined space are shown in Supplementary Fig. 27. As noticed, V_x shows a positive value (Supplementary Fig. 27e), while V_y and V_z exhibit opposite gas flow directions (Supplementary Fig. 27f-g). These results indicate that the backflow gas is generated around the substrate. In other words, no extreme conditions are required to generate the turbulent flow in the inner tube. The turbulent flow is

supposed to enhance the MoS_2 twisted nucleation in the carrier gas because backflows increase the collision rate of molecules to molecules and molecules to substrate by producing enough energy for the twist nucleation, resulting in an alteration of the synthesis conditions. We believe that the introduced backflow gas through the inner tube drives the entire inner tube out of equilibrium to reduce the chance to form more stable 0°- and 60°-TB-MoS_2, which is expected to be the most important reason for the synthesis of TB-MoS_2.

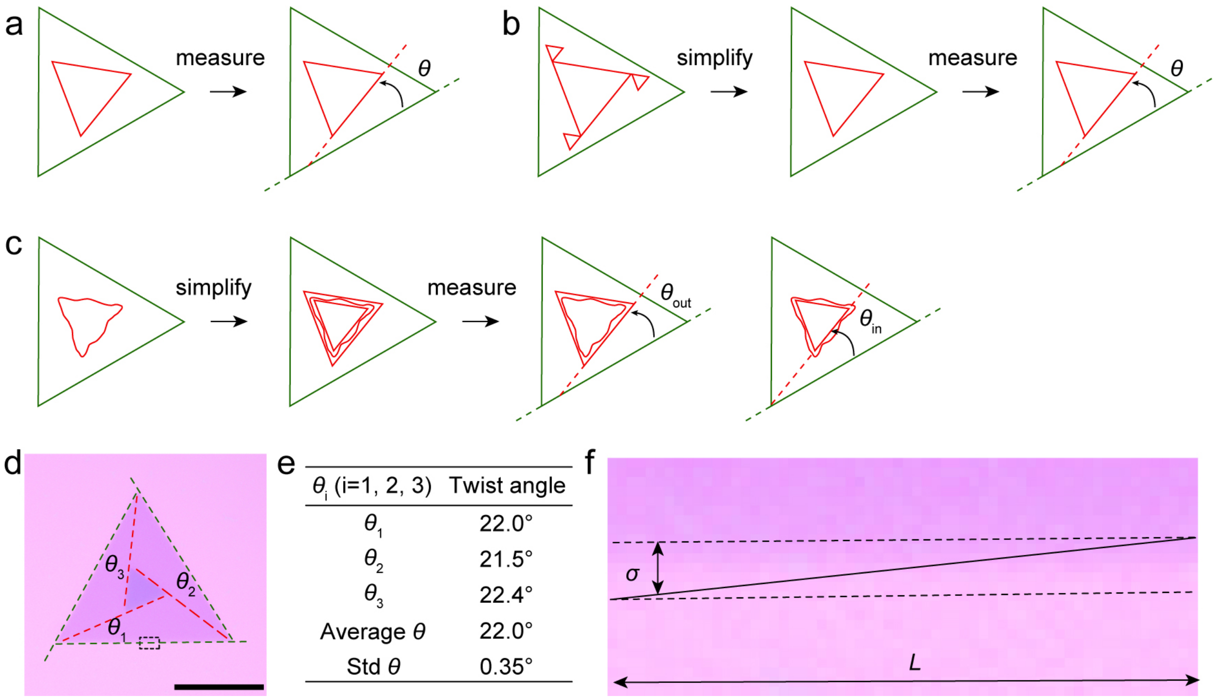

θ_i (i=1, 2, 3)	Twist angle
θ_1	22.0°
θ_2	21.5°
θ_3	22.4°
Average θ	22.0°
Std θ	0.35°

Supplementary Fig. 1 Rules for measuring the twist angles and corresponding error analysis of TB-MoS_2 based on OM method.

a Schematic diagram for measuring the TB-MoS_2 with straight triangle edge (Type I), **b** triangle surrounded by satellite-triangle (Type II), and **c** triangle with irregular edge (Type III). **d** The typical OM image of TB-MoS_2. **e** The measured twist angles based on the OM from the TB- MoS_2 in **d** (scale bar: 20 μm). **f** The origin of error during the measurement.

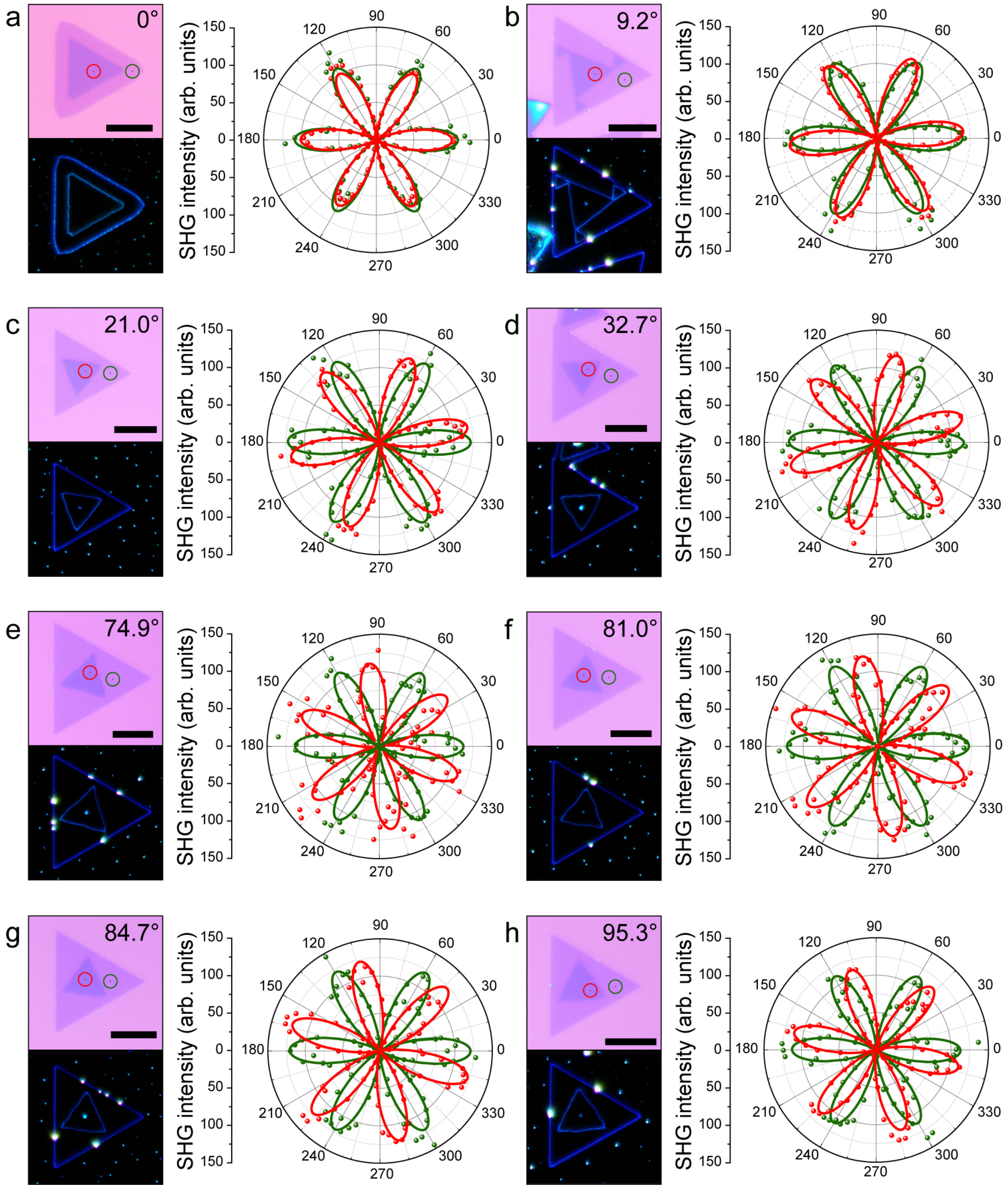

Supplementary Fig. 2 The BF-OM, DF-OM and the corresponding polar plots of parallel components of SHG intensity of TB-MoS_2.

a-h The SHG BF-OM, DF-OM, and SHG results of 0°, 9.2°, 21.0°, 32.7°, 47.9°, 81.0°, 84.7°, and 95.3°-TB-MoS_2, respectively. The corresponding SHG azimuthal angles can be measured to be 0°, 4.5°, 10.5°, 16.3°, 37.3°, 40.3°, 42.5°, 47.7°, respectively. Scale bars: 10 μm.

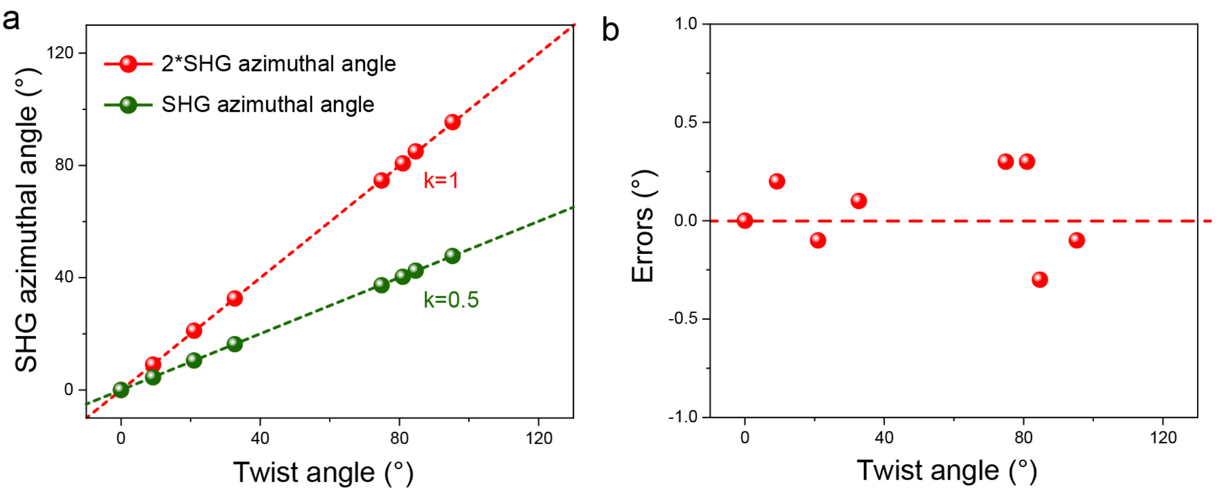

Supplementary Fig. 3 The relationship between the SHG azimuthal angle and twist angle measured from OM in TB-MoS_2.

a The relationship between the azimuthal angle measured from the SHG and the twist angles measured from the OM. **b** The twist angles errors between the twist angle and the twofold SHG azimuthal angle. The SHG azimuthal angle was calculated to be 0°, 4.5°, 10.5°, 16.3°, 37.3°, 40.3°, 42.5°, 47.7° corresponding to the 0°, 9.2°, 21.0°, 32.7°, 47.9°, 81.0°, 84.7°, and 95.3°-TB-MoS_2 samples from Supplementary Fig. 2. The twofold SHG azimuthal is highly consistent with the twist angle measured form OM, which indicated that the accuracy of twist angle measurement from OM.

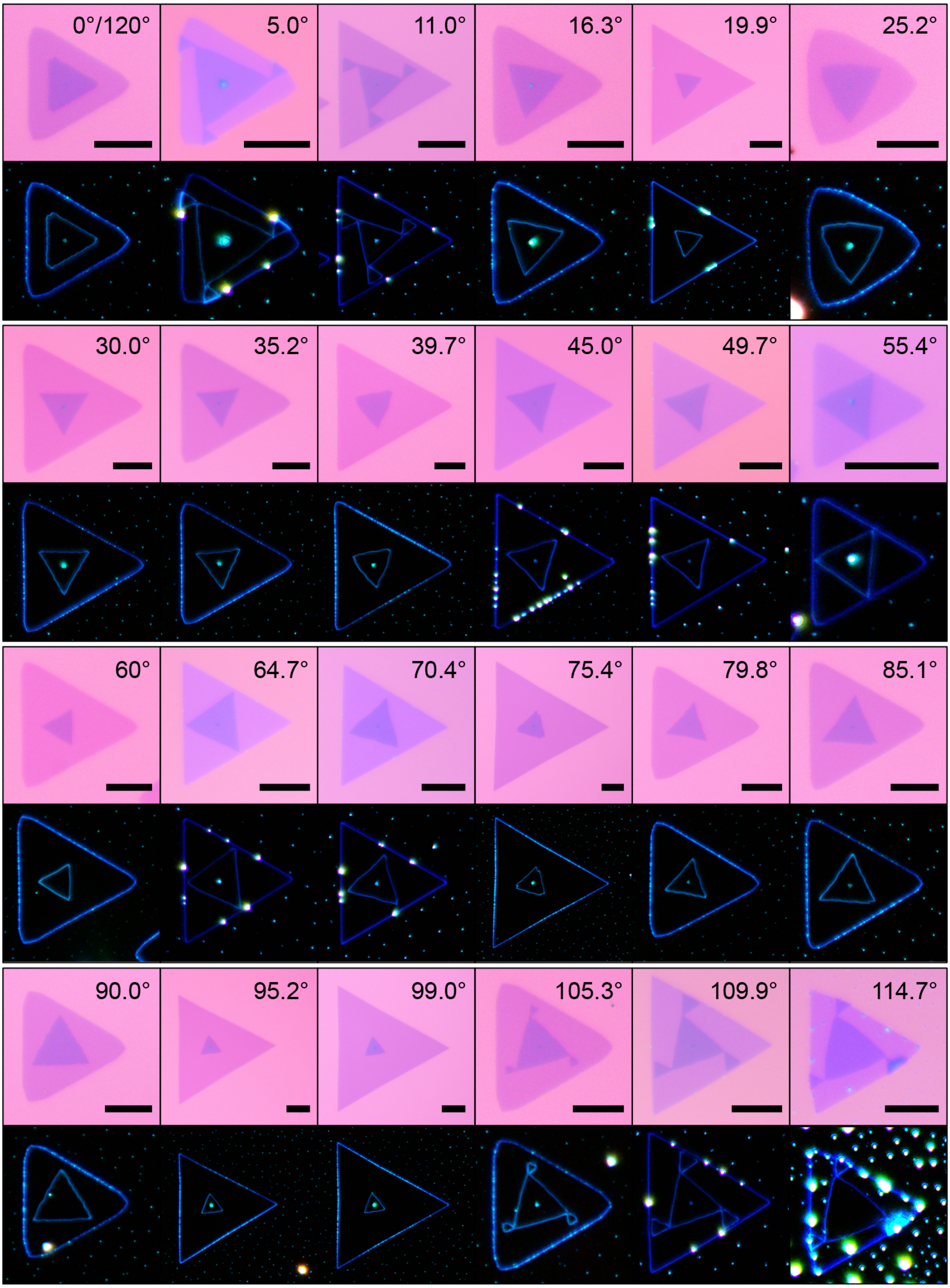

Supplementary Fig. 4 BF-OM and DF-OM images of as-grown TB-MoS_2.

Scale bars: 10 µm.

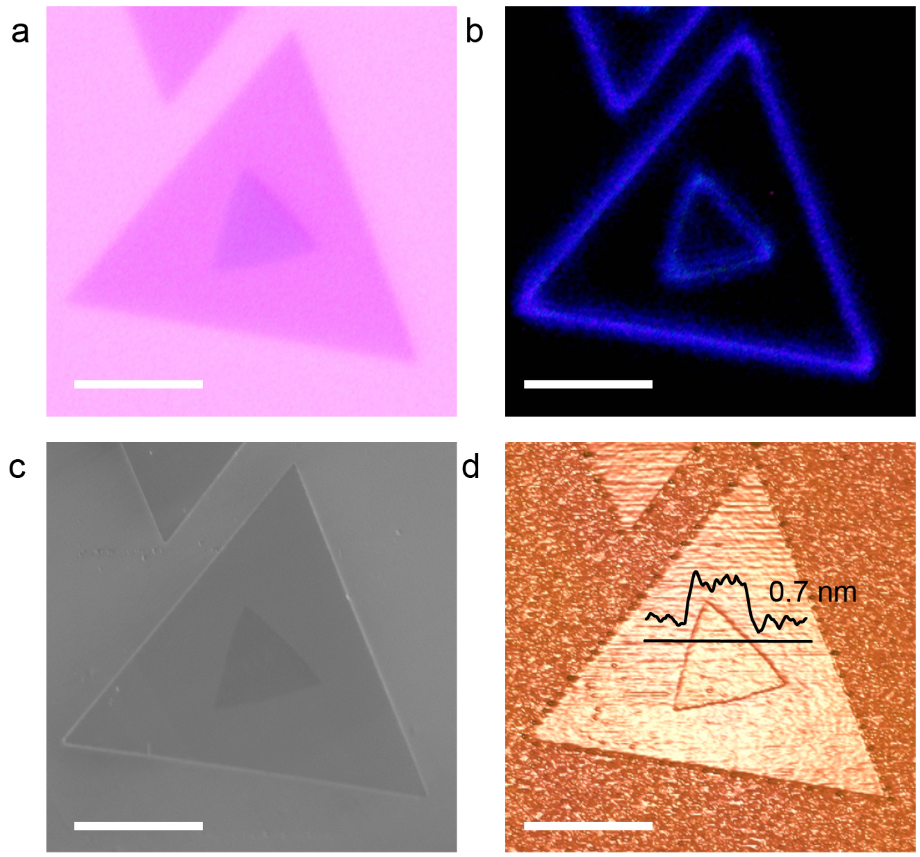

Supplementary Fig. 5 Characterization of 21.9°-TB-MoS_2.

a-d The in-situ BF-OM, DF-OM, SEM, and AFM images of 21.9°-TB-MoS_2. Scale bar: 5 μm.

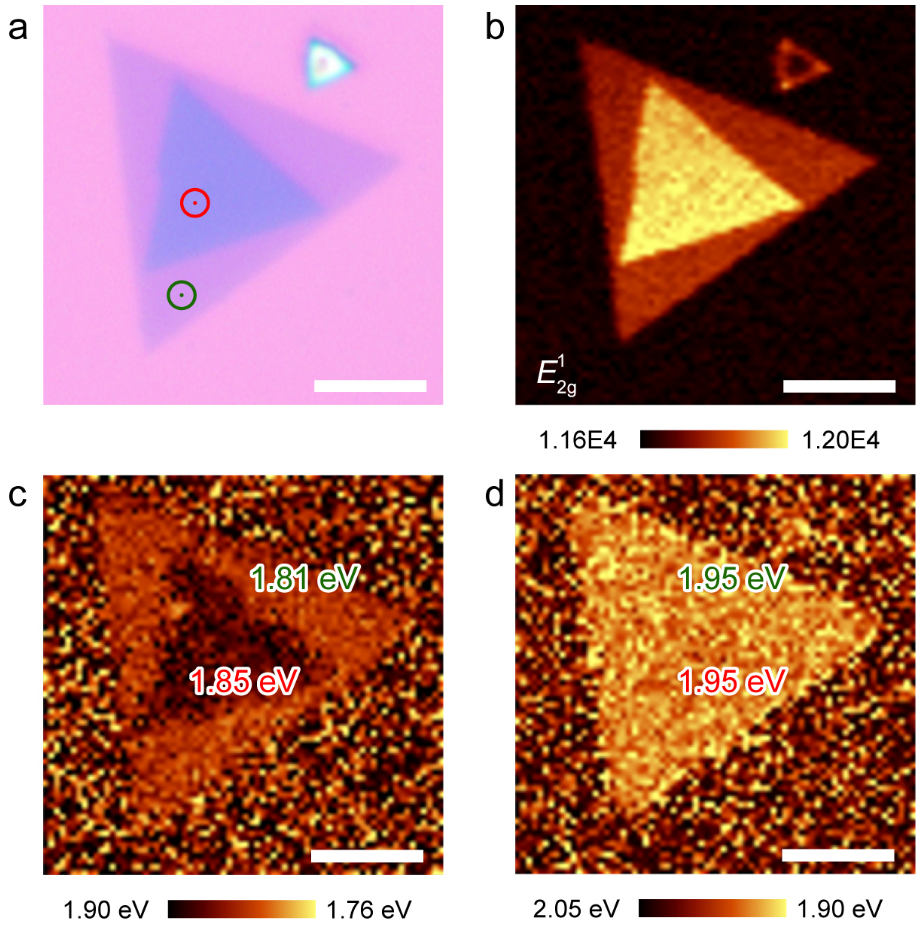

Supplementary Fig. 6 BF-OM image of 101.3°-TB-MoS_2 for Raman and PL mapping.

a The BF-OM image of 101.3°-TB-MoS_2. **b** The Raman intensity mapping for E^1_{2g} mode of 101.3°-TB-MoS_2. **c-d** The PL mapping for position of maximum of 101.3°-TB-MoS_2 in the range around A exciton (1.81 eV) and B exciton (1.95 eV), respectively. Scale bar: 5 μm.

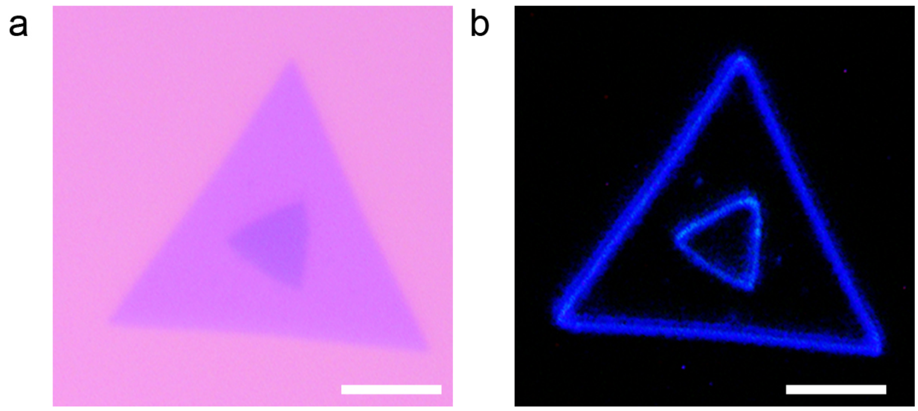

Supplementary Fig. 7 BF-OM image of 90°-TB-MoS_2 for AFM and high-resolution AFM measurement.

Scale bar: 5 µm.

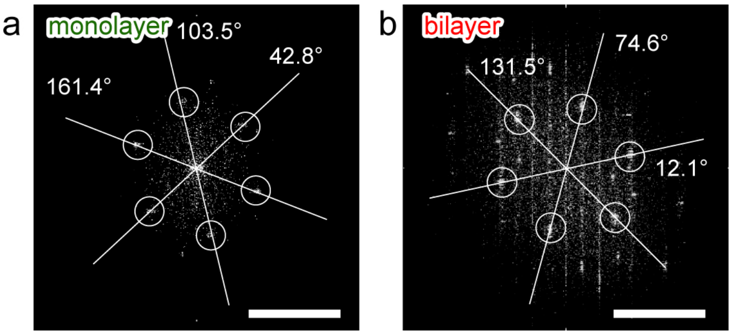

c

$\theta_{Bilayer}$	$\theta_{Monolayer}$	Twist angle
12.1°	42.8°	30.7°
74.6°	103.5°	28.9°
131.5°	161.4°	29.9°
Average		29.8°

Supplementary Fig. 8 The twist angle measurement of 90°-TB-MoS_2 based on the FFT from high-resolution LFM images.

Scale bar: 5 nm^{-1}.

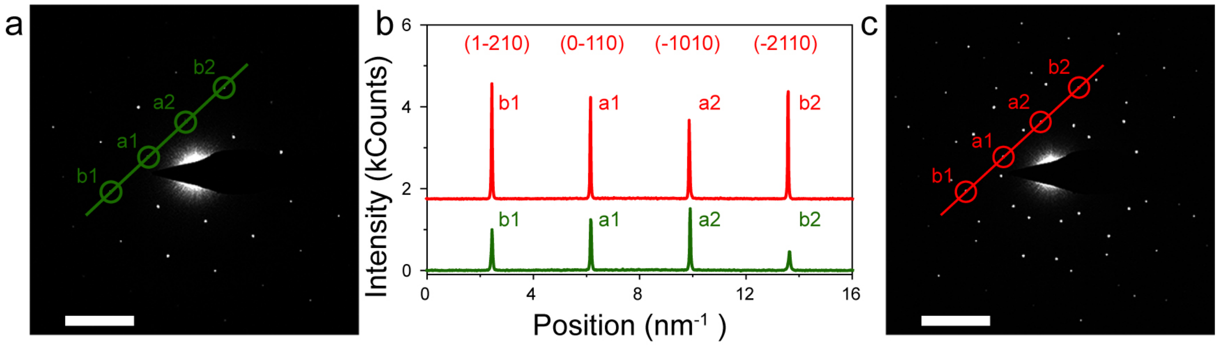

Supplementary Fig. 9 SAED patterns with diffracted intensity of TB-MoS_2.

a SAED patterns of monolayer MoS_2 and **c** TB-MoS_2. **b** The intensity profiles, along the axes marked in **a** and **c** with green and red lines. Scale bar: 5 nm^{-1}.

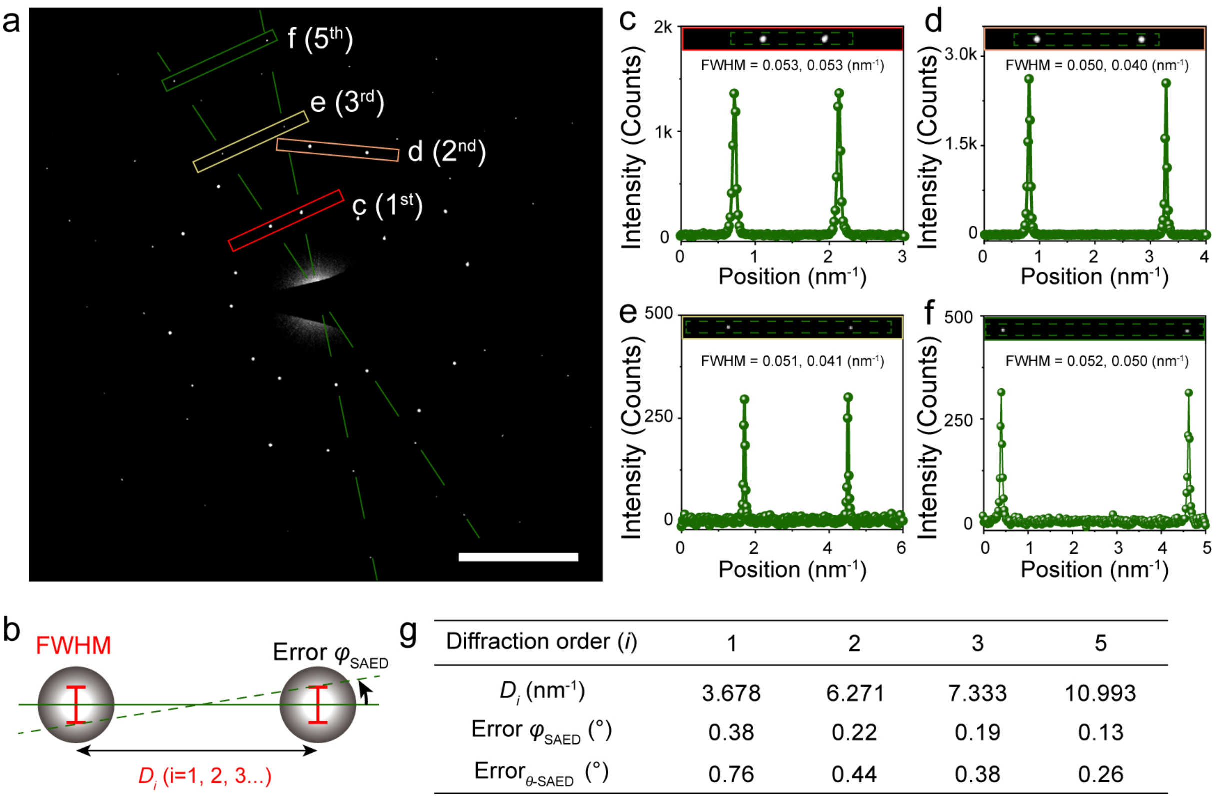

Diffraction order (i)	1	2	3	5
D_i (nm^{-1})	3.678	6.271	7.333	10.993
Error φ_{SAED} (°)	0.38	0.22	0.19	0.13
Error$_{\theta\text{-SAED}}$ (°)	0.76	0.44	0.38	0.26

Supplementary Fig. 10 Error analysis of twist angle measurement based on SAED method.

a Typical SAED pattern of the TB-MoS_2. **b** Illustration of the error determined by the SAED points. **c-f** Position dependent diffraction intensity of four diffraction points shown in **a**. **g** The errors results of SAED method. Scale bar 5 nm^{-1}.

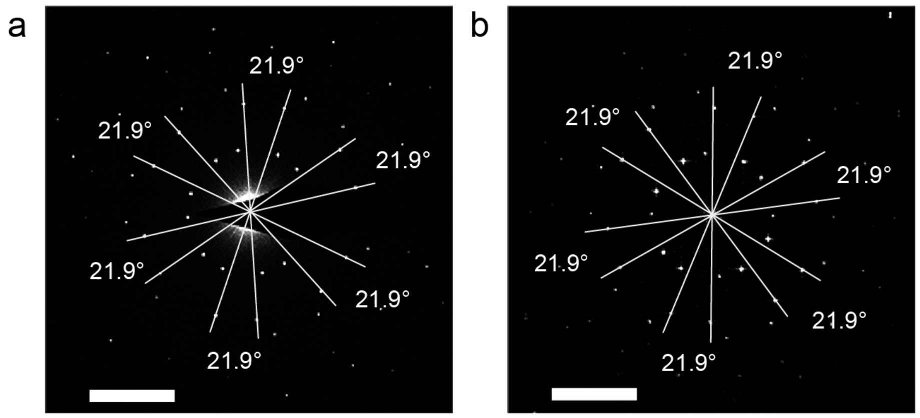

Supplementary Fig. 11 The twist angle measurement of 21.9°-TB-MoS_2.

a SAED pattern and **b** FFT pattern of the TB-MoS_2 in Fig. 2b. The twist angle is calculated as precisely as 21.9°. Scale bar 5 nm^{-1}.

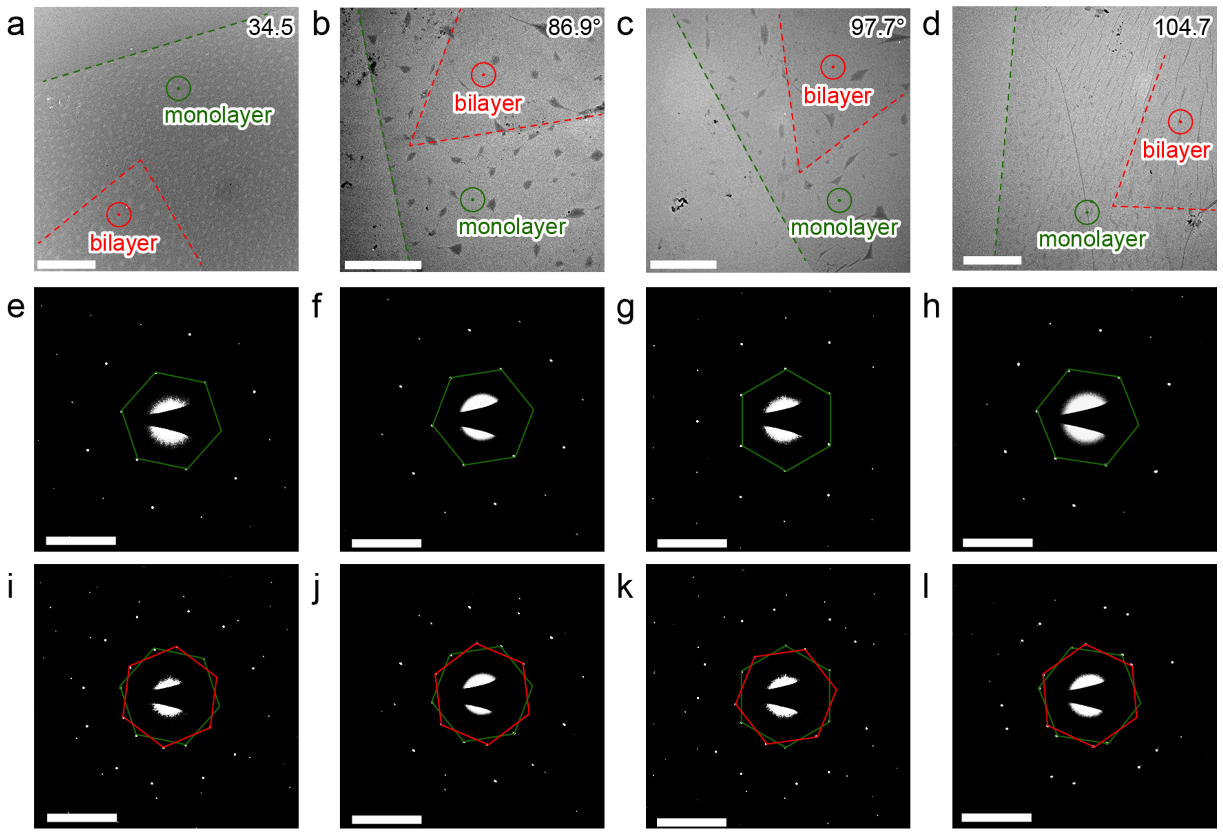

Supplementary Fig. 12 HAADF-STEM results of TB-MoS_2.

a-d TEM-HAADF image of 34.5°, 86.9°, 97.7°, and 104.7°-TB-MoS_2, respectively. **e-h** The SAED patterns of monolayer of 34.5°, 86.9°, 97.7°, and 104.7°-TB-MoS_2 in panel **a-d**, respectively. **i-l** The SAED patterns of bilayer of 34.5°, 86.9°, 97.7°, and 104.7°-TB-MoS_2 in panel **a-d**, respectively. The twist angles (θ_{SAED}) were measured to be 25.5°, 26.9°, 22.3°, and 15.3° form the two adjacent diffraction spots in the SAED of 34.5°, 86.9°, 97.7°, and 104.7°-TB-MoS_2, respectively. Scale bar: 2 μm in **a-d**, 5 nm^{-1} in **e-l**.

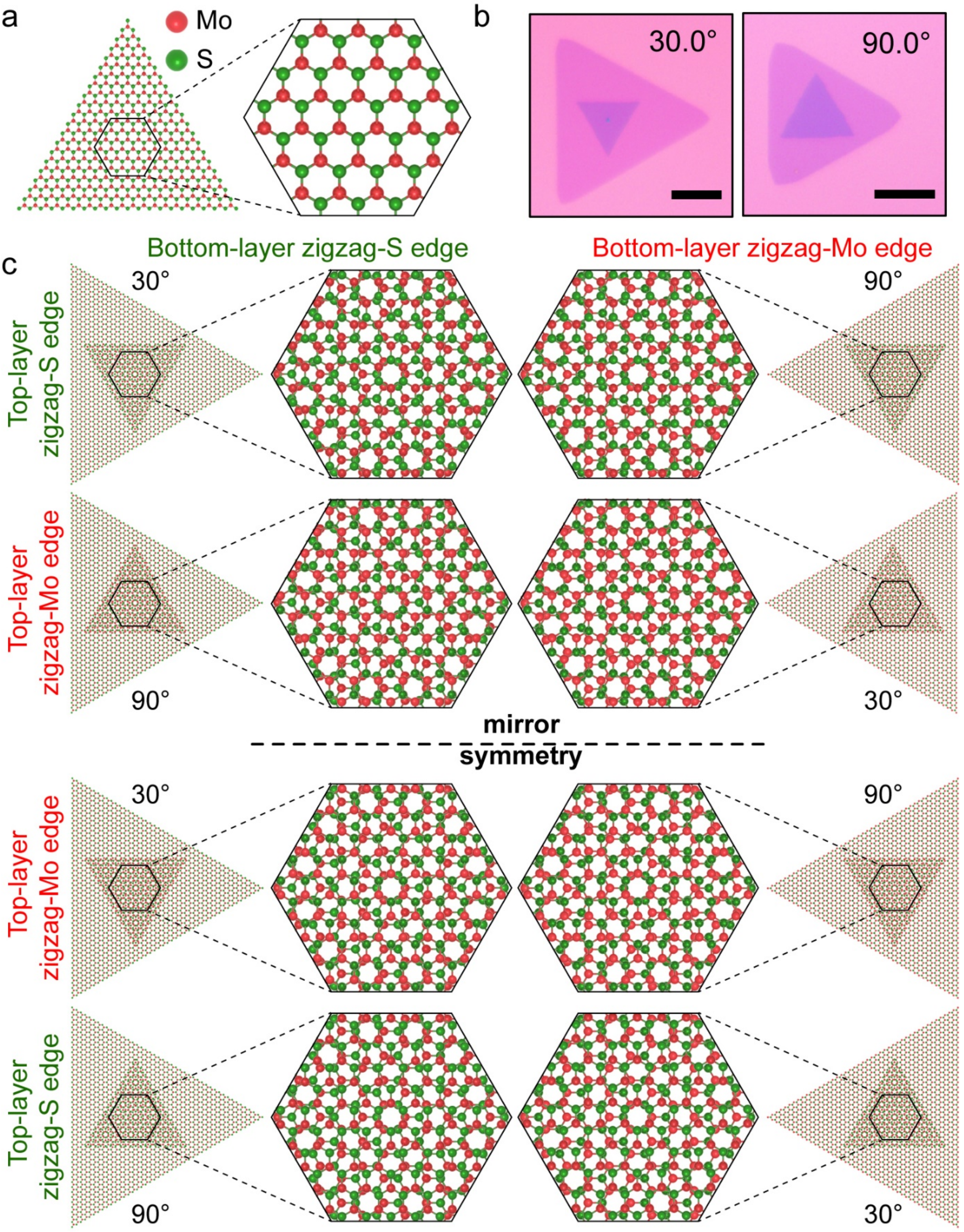

Supplementary Fig. 13 The atom structure of MoS_2.

a Illustration of atomic crystalline structure of twist angle MoS_2.**b** BF-OM and DF-OM of 30.0° and 90.0°-TB-MoS_2, scale bars: 10 μm. **c** The corresponding atomic configuration and enlarged

area 30.0° and 90.0°-TB-MoS_2 with different zigzag-Mo and zigzag-S edge of top and bottom layer, respectively.

As we know, Raman and PL are micro-area analysis methods, wherein only an area of hundreds of nanometers can be detected under a 532 nm laser. Due to the D_{3h}^1 symmetry of monolayer MoS_2 and the D_{3d}^3 symmetry of bilayer MoS_2, the twist angle is optimized from 0°~120° to 0°~60°. As shown in Supplementary Fig. 13a, the triangle MoS_2 shows a three-fold symmetry both in macroscopic OM and hundreds of nanometers area. Three-fold symmetry can also be observed for the macroscopic OM of 30° and 90°-TB-MoS_2 (Supplementary Fig. 13b). It should be noted that the triangle morphology might own the zigzag-S or zigzag-Mo edges, which means eight different combinations (Supplementary Fig. 13c) can be obtained with 30° and 90° twist angle and different zigzag-Mo and zigzag-S edge in bottom and top-layer, respectively. Eight combinations can be classified into two groups with different atom structures. Besides, the two groups of atom structure show mirror symmetry, which indicates that all the 30° and 90°-TB-MoS_2 show the same atom structure or mirror symmetry. Although only 30° and 90°-TB-MoS_2 were discussed, the mirror symmetry can be observed in all the micro-areas of the TB-MoS_2 samples due to the crystal symmetry. Therefore, considering the Raman and PL were measured under the micro-area, the twist angle ($\theta_{\text{Raman-PL}}$) under the Raman and PL can be reduced to 0°~60° based on the following equations by the twist angle (θ_{OM}) measured from OM.

$$\theta_{\text{Raman-PL}} = \theta_{\text{OM}} \left(0 \le \theta_{\text{OM}} \le 60°\right) \tag{14}$$

$$\theta_{\text{Raman-PL}} = 120° - \theta_{\text{OM}} \ \left(60° < \theta_{\text{OM}} \le 120°\right) \tag{15}$$

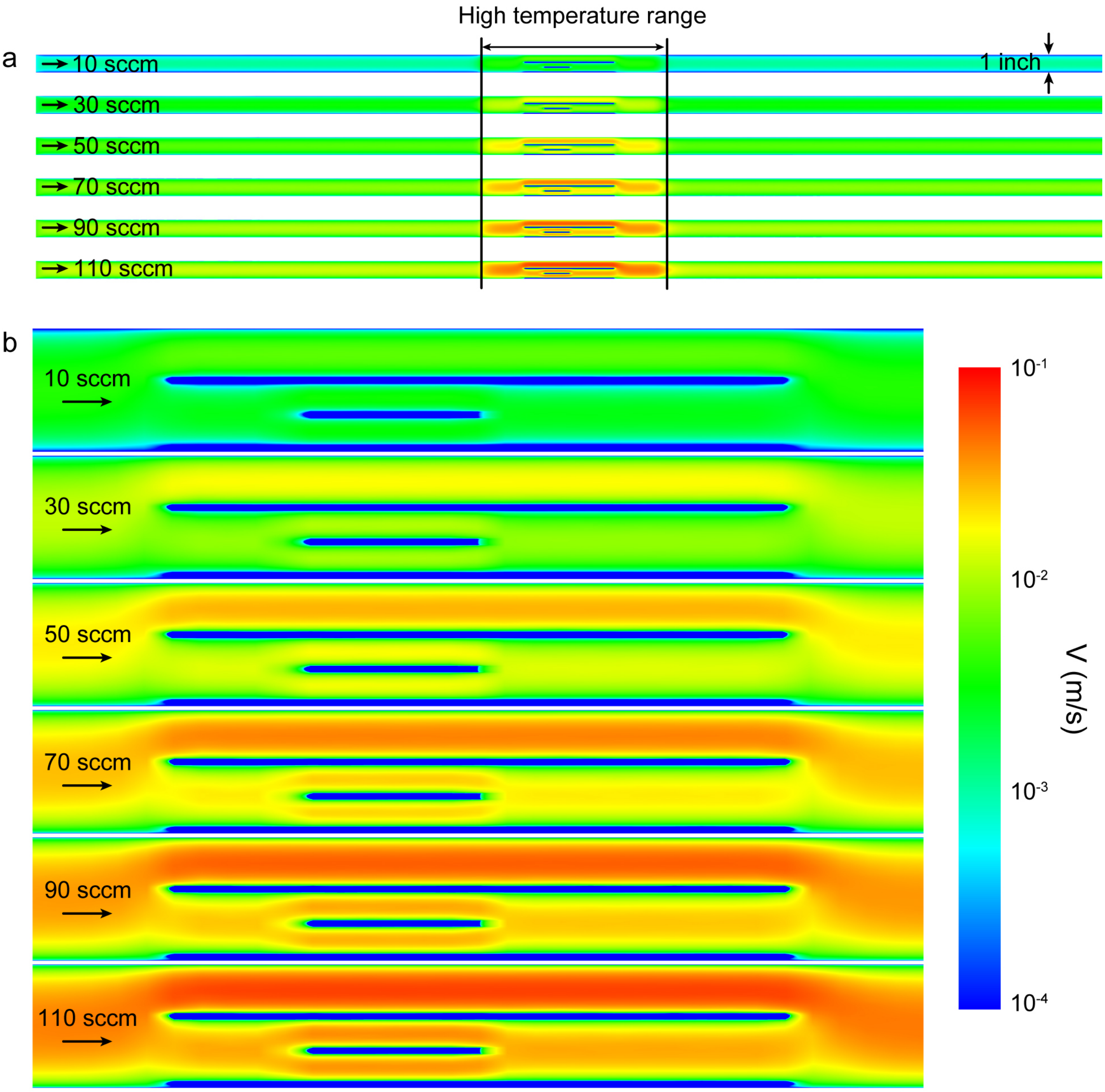

Supplementary Fig. 14 Velocity distribution of the space-confined CVD setup under different gas flow rates.

a The velocity distribution with the small inner-tube under gas flow rates of 10 to 110 sccm, respectively. **b** The enlarged velocity distributions of **a**.

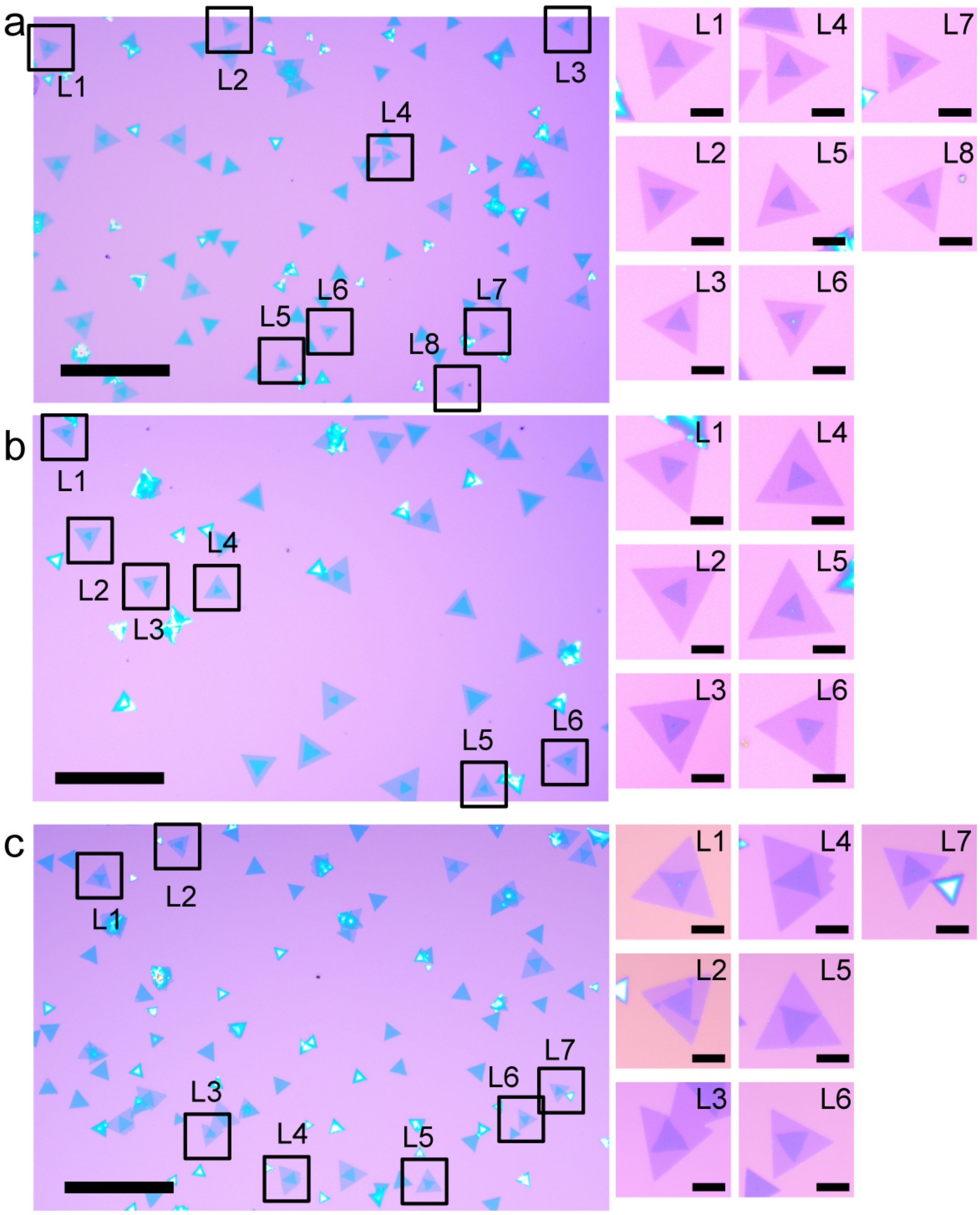

Supplementary Fig. 15 OM of TB-MoS_2 for repeated synthesis in three times.

Scale bars: 100 μm for the OM under 20X objective, 10 μm for the small area under 50X objective. The TB-MoS_2 was repeated synthesis under the fixed gas flow rate of 50 sccm and molar ratio of NaCl to MoO_3 of 20.

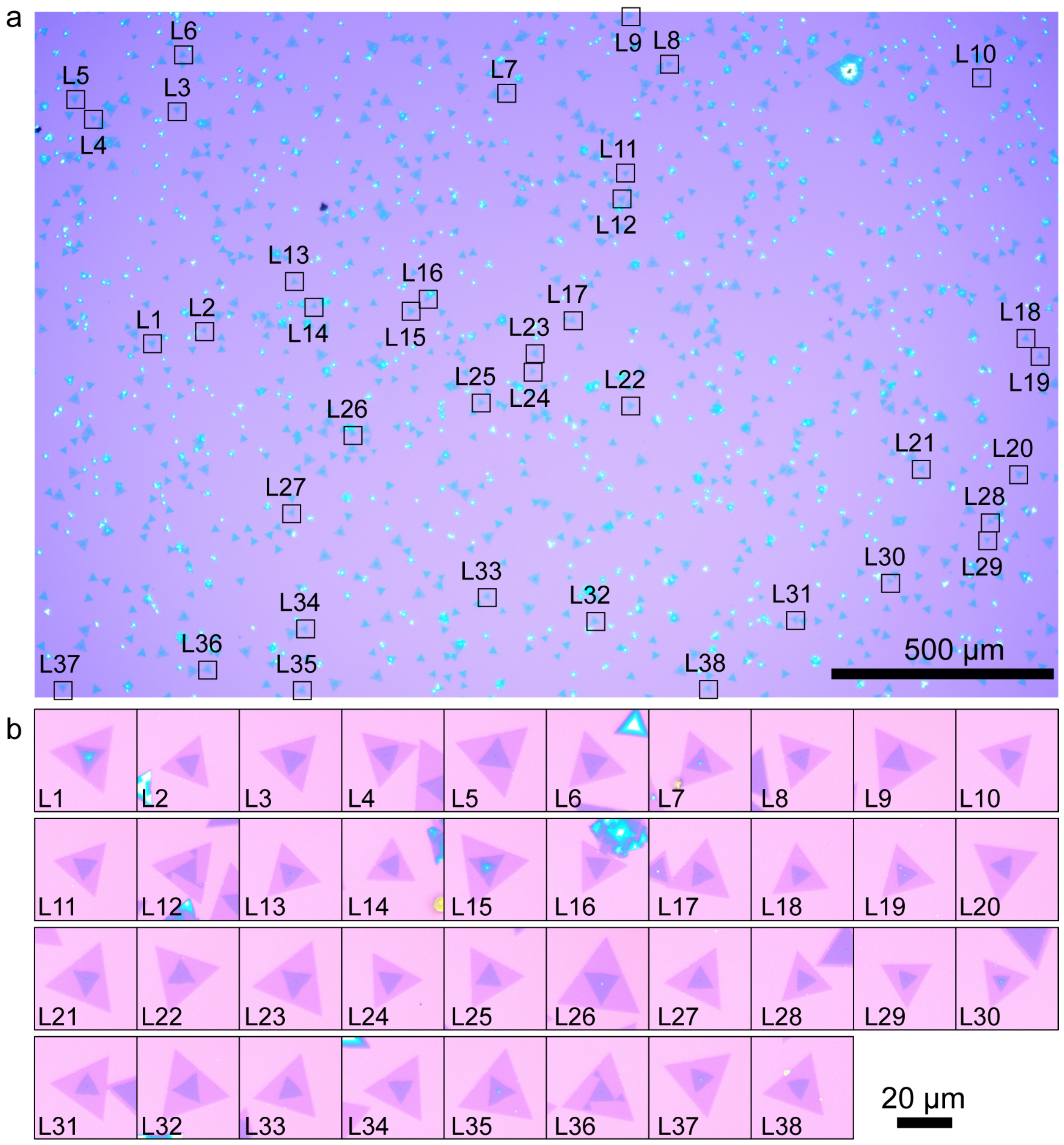

Supplementary Fig. 16 OM of CVD synthesized large-area TB-MoS_2 on SiO_2/Si under the 5X objective.

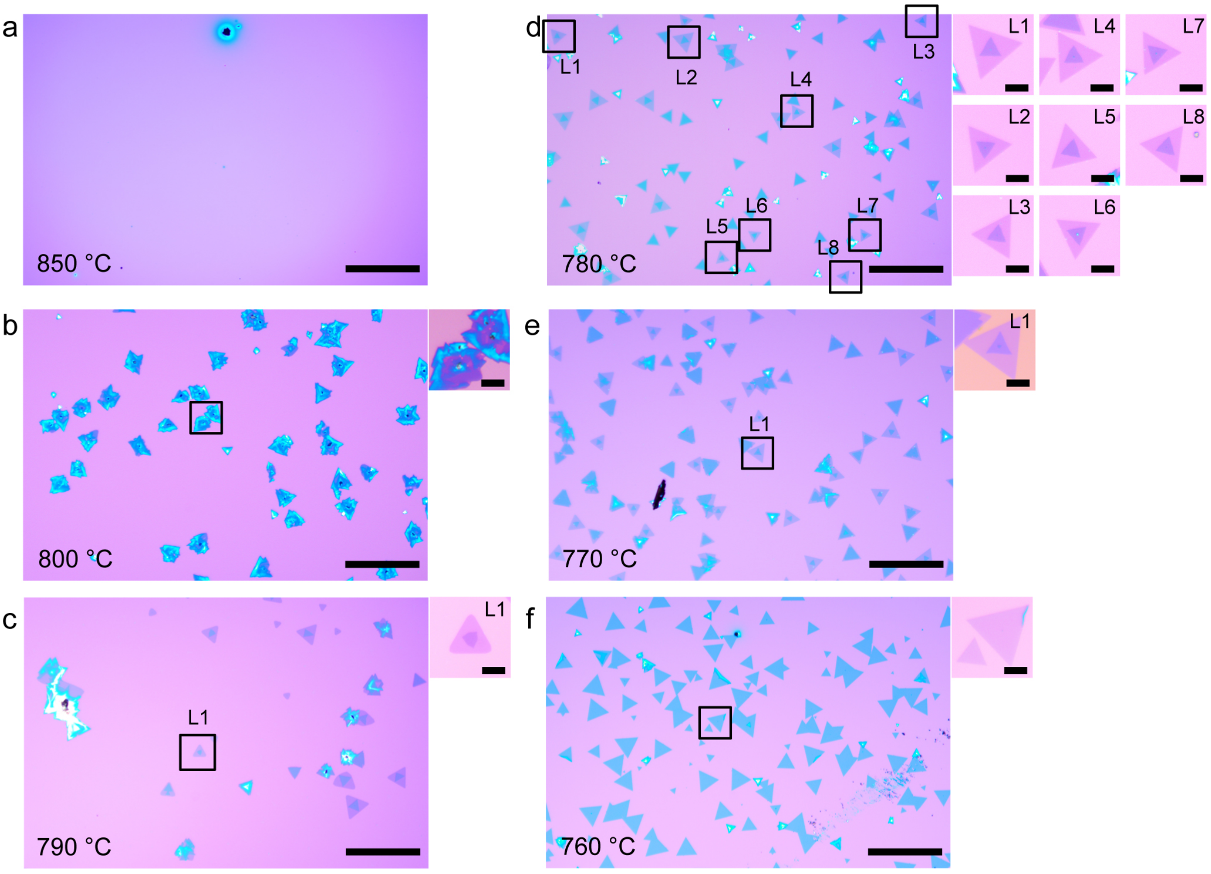

Supplementary Fig. 17 Typical BF-OM of the synthesized products under different reaction temperatures.

a-f Typical OM images of the synthesized MoS_2 under different reaction temperature of 850 °C, 800 °C, 790 °C, 780 °C, 770 °C, and 760 °C. The molar ratio of NaCl to MoO_3 is fixed to 20, and the gas flow rate is fixed to 50 sccm. Scale bars: 100 μm for the OM under 20X objective, 10 μm for the small area under 50X objective.

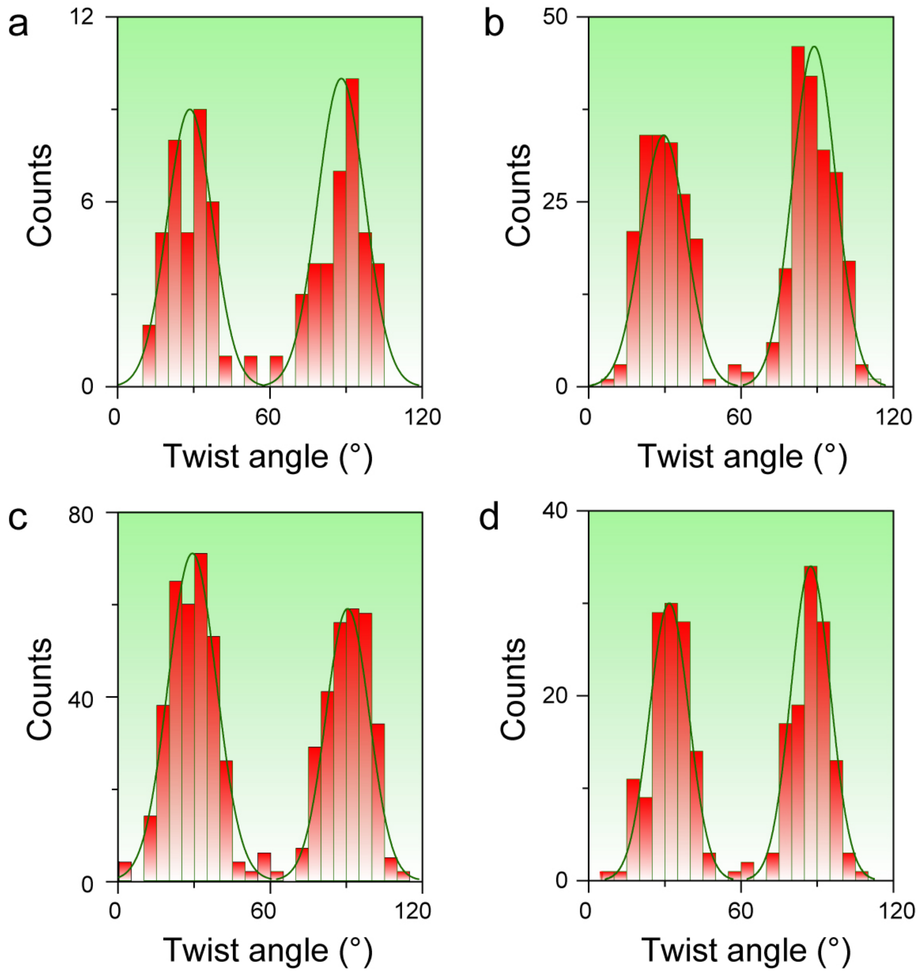

Supplementary Fig. 18 Statistical distribution of twist angles under different growth conditions.

a The TB-MoS_2 synthesized under a gas flow rate of 90 sccm and a NaCl to MoO_3 molar ratio of 20. **b** The TB-MoS_2 synthesized under a gas flow rate of 70 sccm and a NaCl to MoO_3molar ratio of 20. **c** The TB-MoS_2 synthesized under a gas flow rate 30 sccm and a NaCl to MoO_3 molar ratio of 20. **d** The TB-MoS_2 synthesized under a gas flow rate of 50 sccm and a NaCl to MoO_3 molar ratio of 10.

a

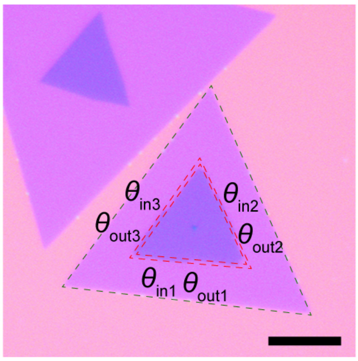

θ_i (i=1, 2, 3)	Twist angle
θ_{in1} / θ_{out1}	1.3° / 1.3°
θ_{in2} / θ_{out2}	1.2° / 1.1°
θ_{in3} / θ_{out3}	1.2° / 1.2°
Average θ	1.2°

b

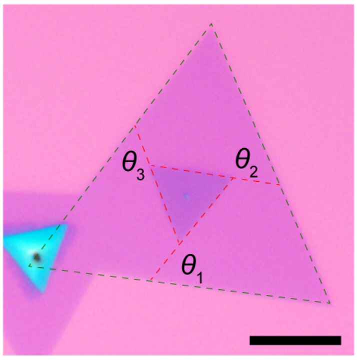

θ_i (i=1, 2, 3)	Twist angle
θ_1	58.2°
θ_2	58.4°
θ_3	58.0°
Average θ	58.2°

Supplementary Fig. 19 OM of synthesized 1.2° and 58.2°-TB-MoS_2.

a-b OM images and corresponding twist angles of 1.2° and 58.2°-TB-MoS_2, respectively. Scale bars: 10 μm.

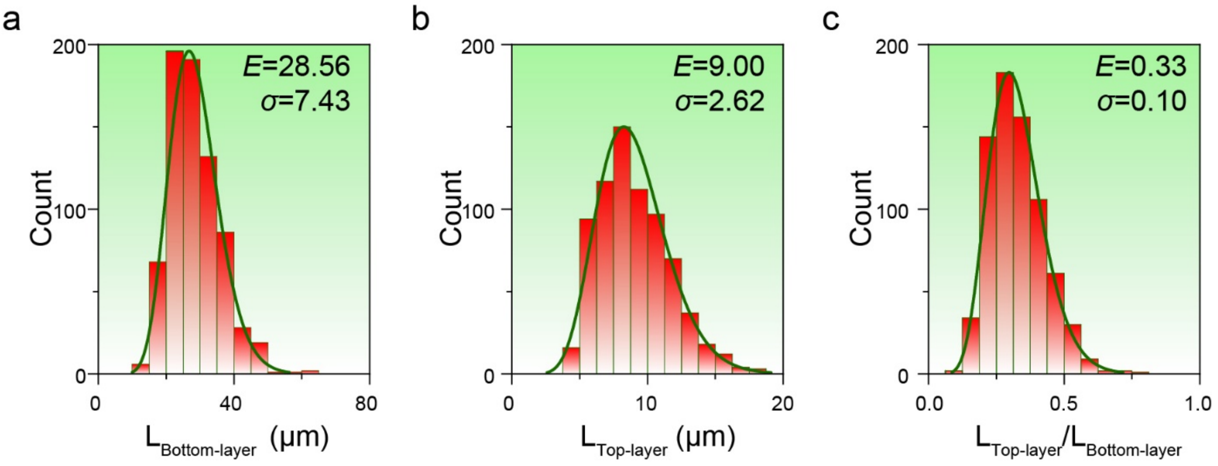

Supplementary Fig. 20 Frequency distribution histograms of flake size of TB-MoS_2 and corresponding gamma distribution fitting curve (with the mean value (*E*) and standard deviation (*σ*)).

a Flake size of bottom-layer in TB-MoS_2. **b** Flake size of top-layer in TB-MoS_2. **c** The bottom-top-layer size ratio in TB-MoS_2.

The frequency distribution histograms of bottom-layer size, top-layer size, and bottom-top-layer size ratio are shown in Supplementary Fig. 20. The gamma distribution fitting curve can well fit all the frequency distribution plots. The average flake size and standard deviation of the bottom-layer in TB-MoS_2 are calculated to be 28.56 μm and 7.43 μm, respectively, which indicates that the size distributes mainly in ~30 μm. This concentrated distribution indicated that our statistics on density are reliable. The flake size of the top-layer in TB-MoS_2 can be calculated to be 9.00 μm, and the bottom-top-layer size ratio in TB-MoS_2 shows a relatively concentrated distribution around 0.33, indicating the uniformity of the samples.

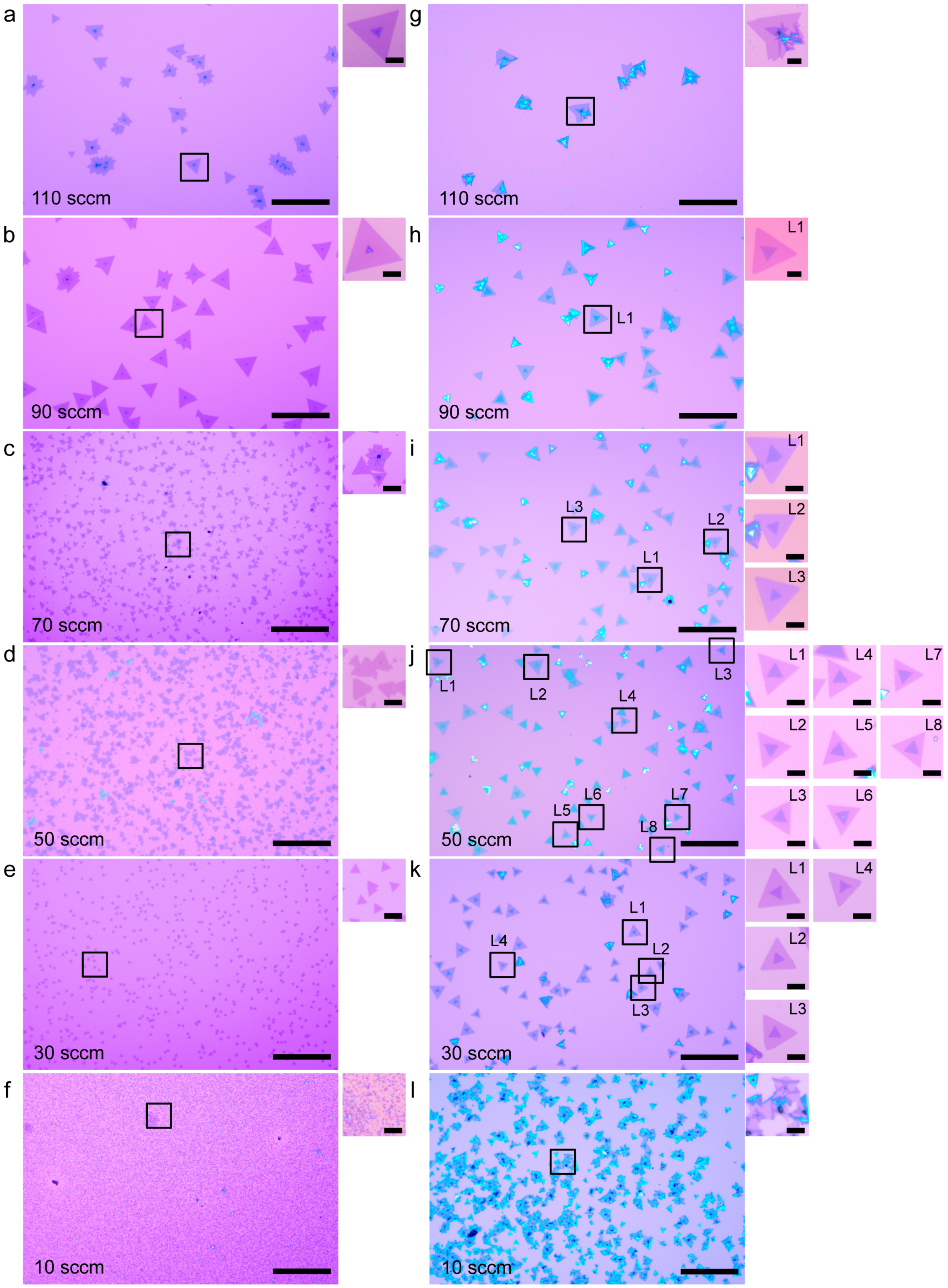

Supplementary Fig. 21 Typical BF-OM of synthesized products under different gas flow rates.

a-f The typical OM synthesized under different gas flow rates without confined space. **g-l** The typical OM synthesized under different gas flow rates with confined space. Scale bars: 100 μm for the OM under 20X objective, 10 μm for the small area under 50X objective. The TB-MoS_2 was synthesized with fixed NaCl to MoO_3 of 20.

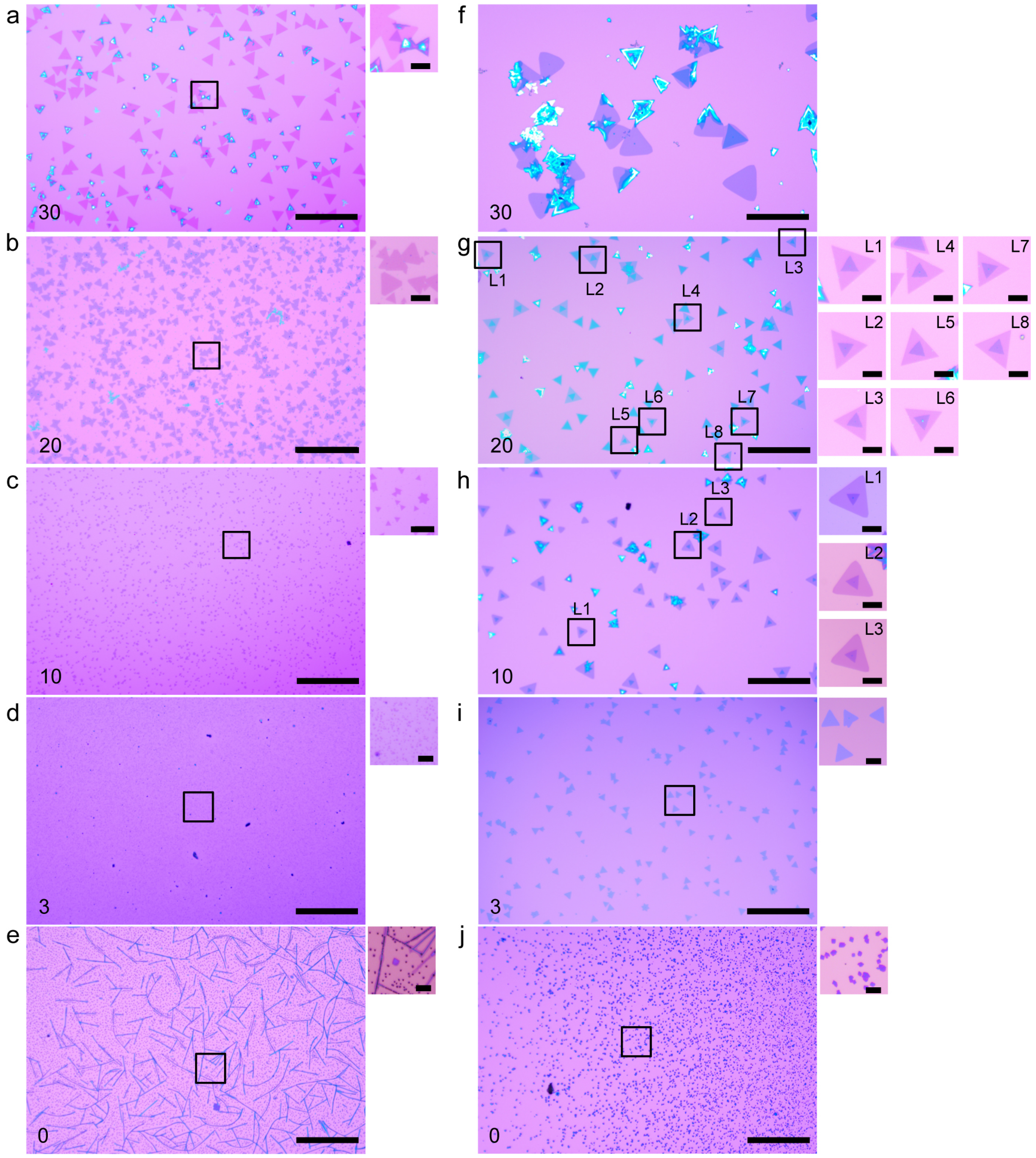

Supplementary Fig. 22 Typical BF-OM of synthesized products under different molar ratios of NaCl to MoO_3.

a-e The typical OM images of the synthesized MoS_2 under different molar ratios of NaCl without confined space. **f-j** The typical OM images of the synthesized MoS_2 under different molar ratios of NaCl with confined space Scale bars: 100 μm for the OM under 20X objective, 10 μm for the small area under 50X objective.

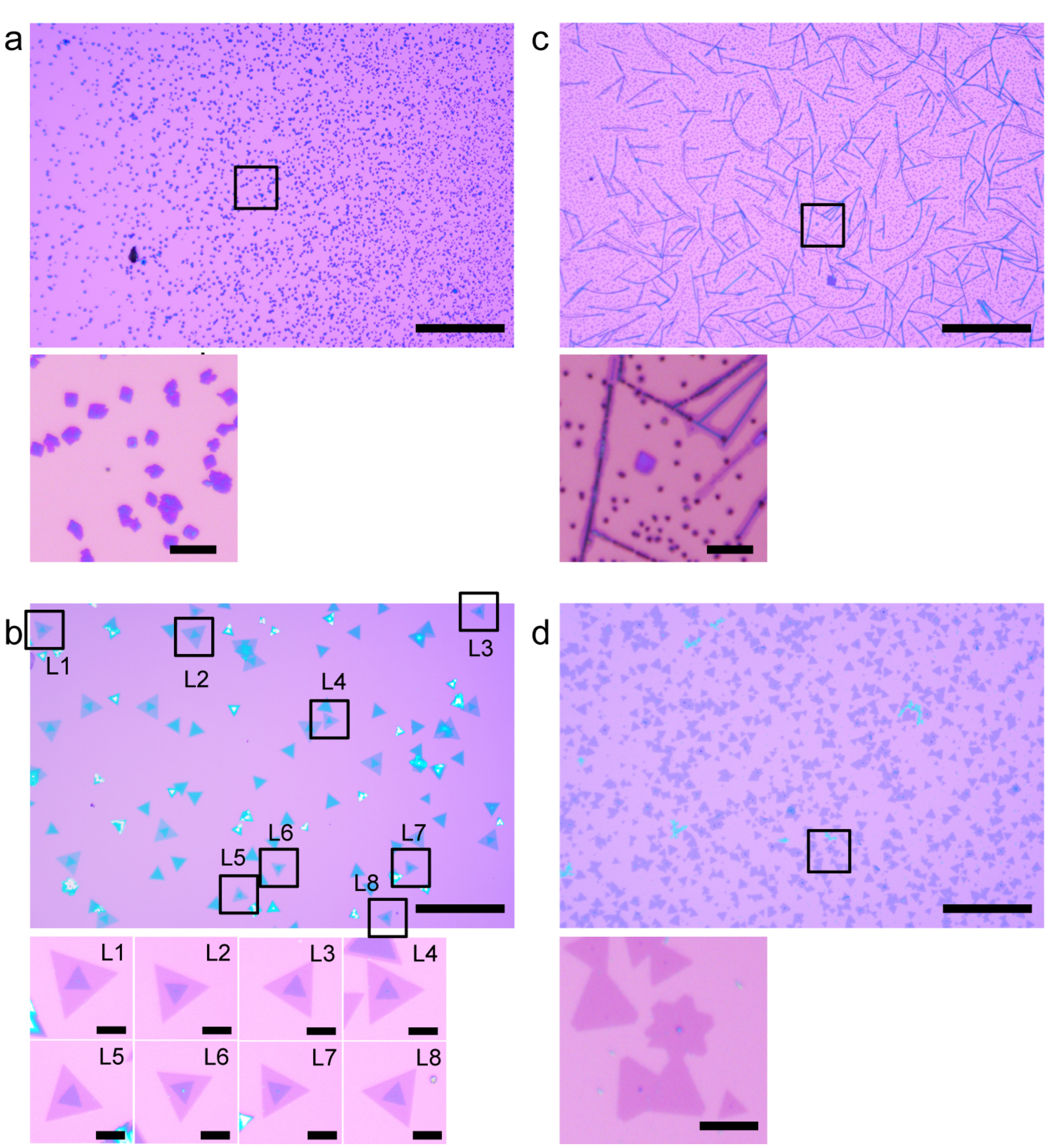

Supplementary Fig. 23 Typical BF-OM of synthesized products with/without NaCl and confined space.

a Without NaCl, with confined space. **b** With NaCl, with confined space. **c** Without NaCl, without confined space. **d** With NaCl, without confined space. Scale bars: 100 µm for the OM under 20X objective, 10 µm for the small area under 50X objective.

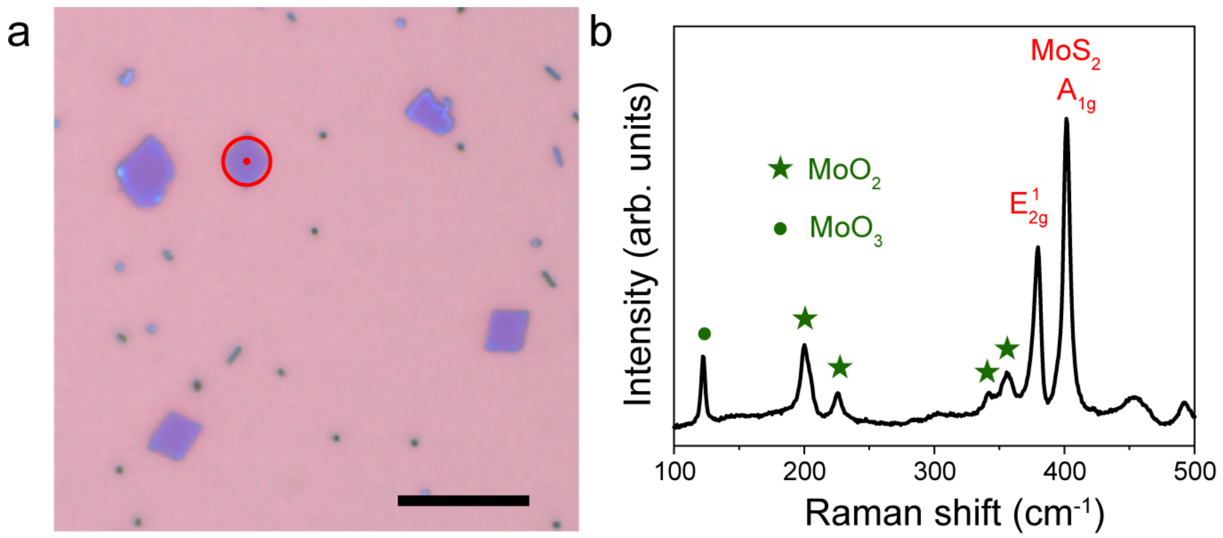

Supplementary Fig. 24 The typical OM and Raman spectrum of the products without adding of NaCl.

a Typical OM and **b** corresponding Raman spectrum of as-synthesized materials without adding NaCl. Scale bars: 10 μm.

The typical OM of as-synthesized materials without adding NaCl is shown in Supplementary Fig. 24a. The corresponding Raman spectrum is taken from the dot in **a**. From the Raman results, it should be noted that the MoS_2 and MoO_3 are concomitant, which indicates that some MoO_3 and MoO_2 have been vulcanized under the high temperature.

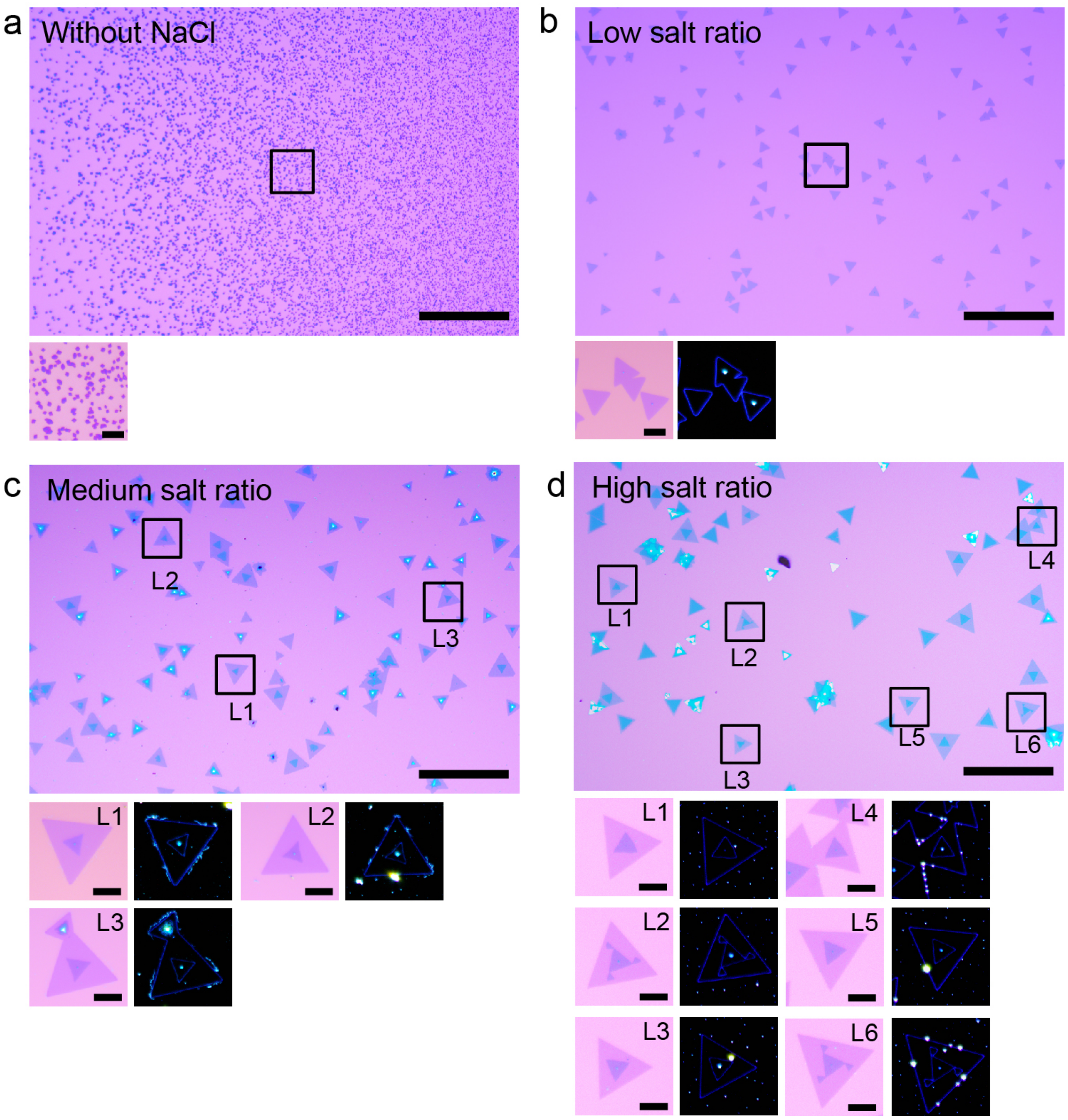

Supplementary Fig. 25 The typical BF-OM of products synthesized under different salt ratio.

a-d The typical OM images of the synthesized materials without NaCl, with low salt ratio, medium salt ratio, and high salt ratio. Scale bars: 100 µm for the OM under 20X objective, 10 µm for the small area under 50X objective. The BF-OM and BF-OM in Supplementary Fig. 25 show the bright dots in the center of the TB-MoS_2, indicating that the TB-MoS_2 was growth from the center nucleation site.

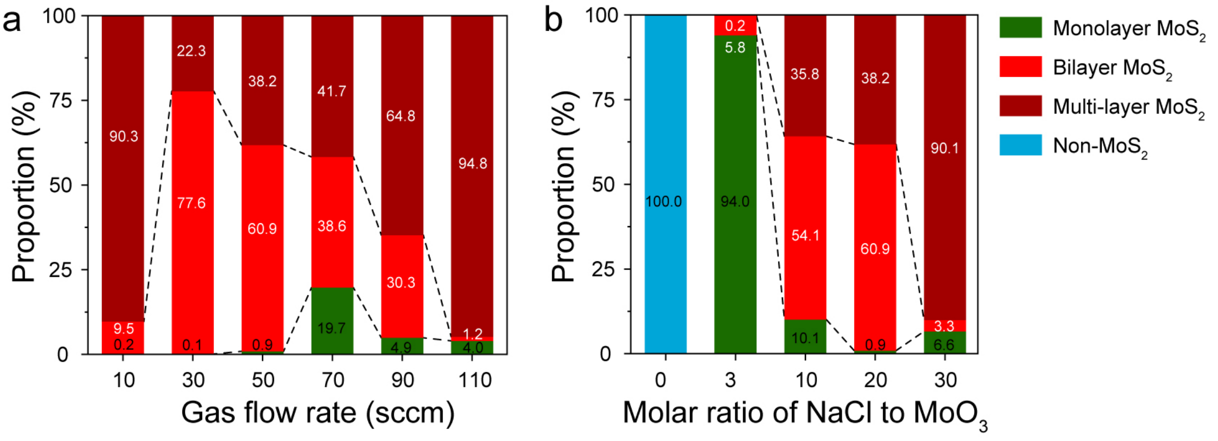

Supplementary Fig. 26 Proportion of monolayer MoS_2, bilayer MoS_2, multi-layer MoS_2, and non-MoS_2 in the as-grown samples.

a-b Under different gas flow rate and molar ratio of NaCl to MoO_3, respectively.

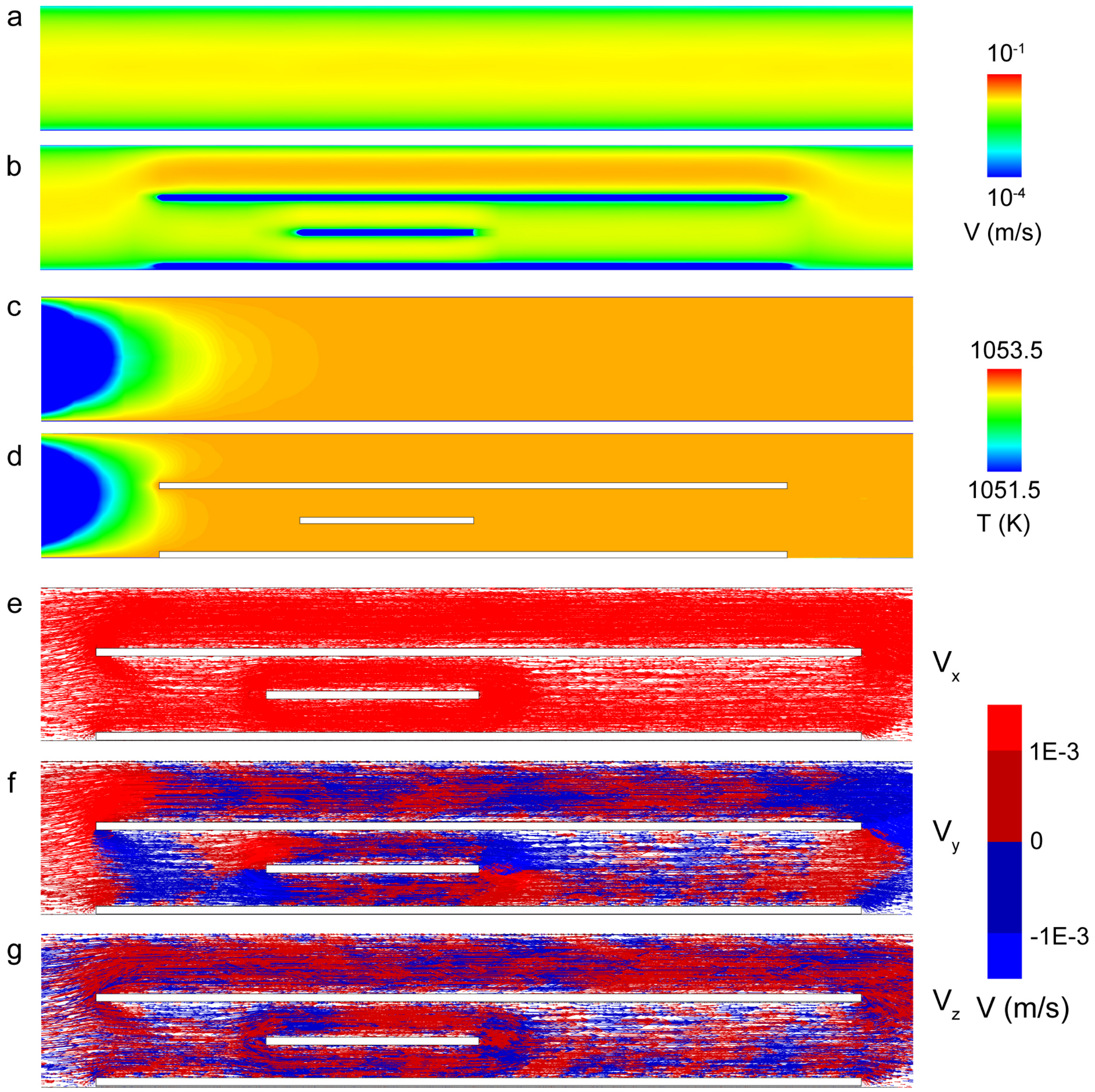

Supplementary Fig. 27 Velocity distribution, temperature distribution, and velocity vector maps of the CVD setup.

a-d Velocity and temperature distribution of the CVD setup with and without confined space, respectively. **e-g** Velocity vector maps of the components of the velocity vector of V_x, V_y, and V_z, respectively. All the results were carried out with a gas flow rate of 50 sccm and a temperature of 780 °C.

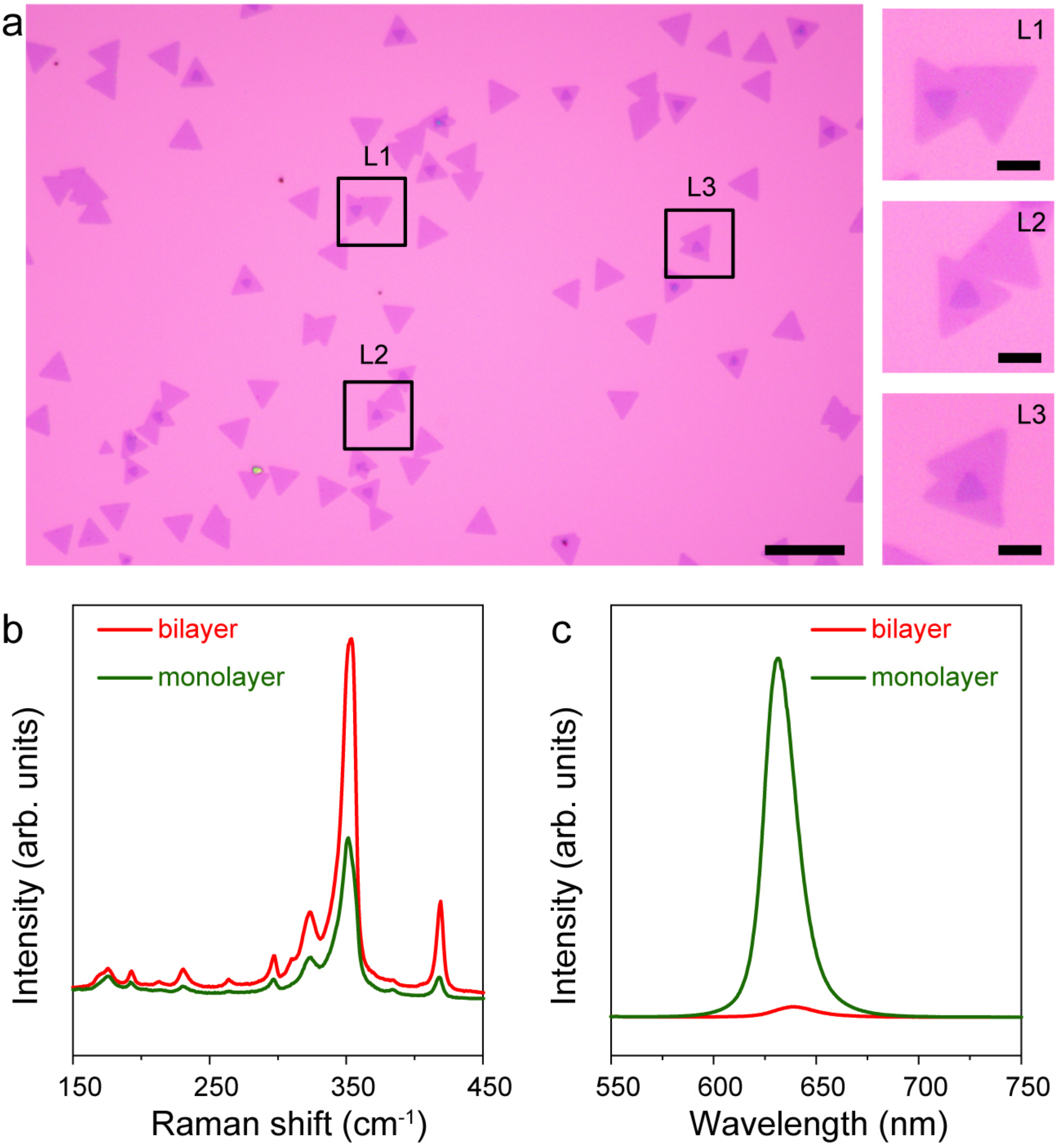

Supplementary Fig. 28 Characterization of TB-WS_2.

a The typical OMs of TB-WS_2. Scale bars: 20 μm for the OM under 50X objective, 5 μm for the single TB-WS_2. **b** Typical Raman spectra and **c** PL spectra of monolayer and 91°-TB-WS_2. The Raman and PL results indicated that the TB-WS_2 has been well synthesized.

Supplementary Table 1. Summary of the CVD preparation of the TB-TMDCs.

Materials	Synthesis parameters of TB-TMDCs			Results of TB-TMDCs				Ref.
	CVD Setup	Precursors	Growth conditions	Twist angle produced	Density	Proportion of bilayer	Yield	
MoS_2	**Conventional method**, 1-inch quartz tube, mica, fused silica, and SiO_2/Si substrate	20 mg MoO_3 in a crucible, 7 mg S in another crucible	Sit at 105 °C with 500 sccm for 1 h, ramp to 700 °C at 15 °C min^{-1} with 10-15 sccm N_2, sit at700 °C for 5-10 min, cool down naturally with 500 sccm N_2	0° to 60°	—	30%	~5%	[11]
MoS_2	**Conventional method,** 1-inch quartz tube, SiO_2/Si substrate	Mo foil, 600 mg S,	T_S=270 °C, T=795 °C, 70 sccm Ar, t=30 min	0° to 60°	—	—	—	[12]
MoS_2	**Conventional method,** SiO_2/Si substrate	8mg NaCl, 20mg MoS_2	900 ℃ for 50 min with 60 sccm Ar/H_2 (95% Ar)	12°	—	—	—	[13]
$MoSe_2$	**Conventional method,** 1-inch quartz tube, SiO_2/Si substrate	MoO_3, Se powder	T_{Se}=300 °C, T=700 °C, 65 sccm Ar and 5 sccm H_2, t=10 min	θ =7°, 21°, 25°	—	—	—	[14]
WS_2	**Conventional method,** two-temperature-zone tube, quartz substrate	WO_3 powder, 0.1 g S powder	200 sccm Ar for 30 min before heating, 20 sccm Ar for ramping to 1100 °C at the rate of 20 °C min^{-1}, growing for 20 min	θ = 13°, 30°, 41°, 83°	—	—	—	[15]
WS_2	**Conventional method,** SiO_2/Si substrate	10 mg SnO_2, 5 mg NaCl, 100 mg WO_3, 480 mg S powder	180 sccm Ar for 10 min before heating, 180 sccm Ar and 15 sccm H_2 for ramping to 810 °C in 45 min, growing for 3 min	θ = 10°, 20°, 31°, 36°, 40°, 55°	—	90%	—	[16]
				40°	—	—	—	[17]
WSe_2	**Conventional method,** SiO_2/Si substrate	30 mg WO_3, 10 mg SnO_2, 100 mg Se	810 ℃ for 10 min with Ar and H_2	θ = 1.5°, ~24°, ~30°	—	—	—	[18]
**MoS_2**	**Space-confined method, 1-inch quartz tube, SiO_2/Si substrate**	**0.8 mg mixed MoO_3/NaCl, sufficient S powder**	**600 sccm for 10 min before heating, 50 sccm Ar for ramping to 780 °C at a rate of 30 °C/min, growing for 5 minutes**	**0° to 120 ° (3.8% for small twist angles)**	**28.9 pieces/ mm^2**	**60.9%**	**17.2%**	**Our work**

Proportion of bilayer: The proportion of bilayer TMDCs in all obtained TMDCs domains.

Yield: The proportion of TB-TMDCs in the bilayer TMDCs.

Supplementary References

1. Zhou J. D., *et al.* A library of atomically thin metal chalcogenides. *Nature* **556**, 355-359 (2018).

2. Xu M. Z., *et al.* Machine Learning Driven Synthesis of Few-Layered WTe_2 with Geometrical Control. *J. Am. Chem. Soc.* **143**, 18103-18113 (2021).

3. Wang J. W., *et al.* Twin Defect Derived Growth of Atomically Thin MoS_2 Dendrites. *ACS Nano* **12**, 635-643 (2018).

4. Zhang X. M., *et al.* Transition metal dichalcogenides bilayer single crystals by reverse-flow chemical vapor epitaxy. *Nat. Commun.* **10**, 598 (2019).

5. Meng L. J., *et al.* Anomalous thickness dependence of Curie temperature in air-stable two-dimensional ferromagnetic 1T-$CrTe_2$ grown by chemical vapor deposition. *Nat. Commun.* **12**, 809 (2021).

6. Shao G. L., *et al.* Shape-Engineered Synthesis of Atomically Thin 1T-SnS_2 Catalyzed by Potassium Halides. *ACS Nano* **13**, 8265-8274 (2019).

7. Liu L., *et al.* Uniform nucleation and epitaxy of bilayer molybdenum disulfide on sapphire. *Nature* **605**, 69-75 (2022).

8. Feldman Y., Wasserman E., Srolovitz D. J., Tenne R. High-Rate, Gas-Phase Growth of MoS_2 Nested Inorganic Fullerenes and Nanotubes. *Science* **267**, 222-225 (1995).

9. Goto M., *et al.* Synthesis of Epitaxial MoS_2/MoO_2 Core–Shell Nanowires by Two-Step Chemical Vapor Deposition with Turbulent Flow and Their Physical Properties. *ACS Omega* **7**, 39362-39369 (2022).

10. Sun L. Z., *et al.* Hetero-site nucleation for growing twisted bilayer graphene with a wide range of twist angles. *Nat. Commun.* **12**, 2391 (2021).

11. Liu K. H., *et al.* Evolution of interlayer coupling in twisted molybdenum disulfide bilayers. *Nat. Commun.* **5**, 4966 (2014).

12. Han J. L., Cao F., Ji X. H. Formation mechanism and twist-angle dependent optical properties of bilayer MoS_2 grown by chemical vapor deposition. *CrystEngComm* **23**, 2889-2896 (2021).

13. Liu W. Y.*, et al.* Inversion Symmetry and Exotic Interlayer Exciton Behavior in Twisted Trilayer MoS_2 Produced by Vapor Deposition. *ACS Appl. Mater. Interfaces* **15**, 4724-4732 (2023).

14. Bachmatiuk A., Abelin R. F., Quang H. T., Trzebicka B., Eckert J., Rummeli M. H. Chemical vapor deposition of twisted bilayer and few-layer $MoSe_2$ over SiO_x substrates. *Nanotechnology* **25**, 365603 (2014).

15. Zheng S. J.*, et al.* Coupling and Interlayer Exciton in Twist-Stacked WS_2 Bilayers. *Adv. Opt. Mater.* **3**, 1600-1605 (2015).

16. Shao G. L.*, et al.* Twist Angle-Dependent Optical Responses in Controllably Grown WS_2 Vertical Homojunctions. *Chem. Mater.* **32**, 9721-9729 (2020).

17. Zhao S. W., Shao G. L., Han Z. V., Liu S., Zhang T. Y. Gate tunable spatial accumulation of valley-spin in chemical vapor deposition grown 40°-twisted bilayer WS_2. *Journal of Semiconductors* **44**, 012001 (2023).

18. Zheng H. H.*, et al.* Strong Interlayer Coupling in Twisted Transition Metal Dichalcogenide Moiré Superlattices. *Adv. Mater.* **35**, 2210909 (2023).
